# The miR-15b-Smurf2-HSP27 axis promotes pulmonary fibrosis

**DOI:** 10.1186/s12929-023-00896-5

**Published:** 2023-01-07

**Authors:** Seulgi Jeon, Hee Jin, Jin-Mo Kim, Youmin Hur, Eun Joo Song, Yoon-Jin Lee, Younghwa Na, Jaeho Cho, Yun-Sil Lee

**Affiliations:** 1 Graduate School of Pharmaceutical Sciences, Ewha Womans University, 52, Ewhayeodae-gil, Seodaemun-gu, Seoul, 03760 Republic of Korea; 2 Inhalation Toxicity Research Group, Korea Institute of Toxicology, Jeongeup-si, Jeollabuk-do 56212 Republic of Korea; 3 Department of Radiation Oncology, Yonsei University Health System, 50, Yonsei-ro, Seodaemun-gu, Seoul, 03722 Republic of Korea; 4 Department of Manufacturing Pharmacy, Natural Product Research Institute, College of Pharmacy, Seoul National University, Seoul, 08826 Republic of Korea; 5 Korea Institute of Radiological and Medical Science, Seoul, 01812 Republic of Korea; 6 College of Pharmacy, CHA University, 120, Haeryong-ro, Pocheon-si, Gyeonggi-do 11160 Republic of Korea

**Keywords:** HSP27, Phosphorylation, miRNA, Pulmonary fibrosis, Smurf2, Protein degradation

## Abstract

**Background:**

Heat shock protein 27 (HSP27) is overexpressed during pulmonary fibrosis (PF) and exacerbates PF; however, the upregulation of HSP27 during PF and the therapeutic strategy of HSP27 inhibition is not well elucidated.

**Methods:**

We have developed a mouse model simulating clinical stereotactic body radiotherapy (SBRT) with focal irradiation and validated the induction of RIPF. HSP25 (murine form of HSP27) transgenic (TG) and LLC1-derived orthotropic lung tumor models were also used. Lung tissues of patients with RIPF and idiopathic pulmonary fibrosis, and lung tissues from various fibrotic mouse models, as well as appropriated cell line systems were used. Public available gene expression datasets were used for therapeutic response rate analysis. A synthetic small molecule HSP27 inhibitor, J2 was also used.

**Results:**

HSP27 expression with its phosphorylated form (pHSP27) increased during PF. Decreased mRNA expression of SMAD-specific E3 ubiquitin-protein ligase 2 (Smurf2), which is involved in ubiquitin degradation of HSP27, was responsible for the increased expression of pHSP27. In addition, increased expression of miRNA15b was identified with decreased expression of Smurf2 mRNA in PF models. Inverse correlation between pHSP27 and Smurf2 was observed in the lung tissues of PF animals, an irradiated orthotropic lung cancer models, and PF tissues from patients. Moreover, a HSP27 inhibitor cross-linked with HSP27 protein to ameliorate PF, which was more effective when targeting the epithelial to mesenchymal transition (EMT) stage of PF.

**Conclusions:**

Our findings identify upregulation mechanisms of HSP27 during PF and provide a therapeutic strategy for HSP27 inhibition for overcoming PF.

**Supplementary Information:**

The online version contains supplementary material available at 10.1186/s12929-023-00896-5.

## Background

Pulmonary fibrosis (PF) is a chronic and fatal interstitial lung disease involving deposition of collagen and extracellular matrix (ECM) around the fibrotic foci, as well as honeycomb changes of the subpleural and basement membranes. The most common type of PF is idiopathic pulmonary fibrosis (IPF), with a median survival of only 2–4 years [1]. Recently, the incidence rate of a persistent lung damage was shown to surpass 30% in patients being treated for moderate COVID, and approximately 30% of them have severe fibrotic lung damage [2]. Unfortunately, the pathogenesis of PF is poorly understood, and there are no effective therapeutic targets and drugs.

HSP27 (HSP27 in humans and HSP25 in mice) is an ATP-independent molecular chaperone and is a critical mediator in cancer progression, migration and invasion [3], and drug resistance in cancers [4–6]. Recently, HSP27 has been reported as an inducer of the epithelial to mesenchymal transition (EMT) during PF [7, 8]. HSP27 gene silencing by OGX-427, a second-generation antisense oligonucleotide, or direct injection of siHSP27 to the lung inhibited bleomycin (BLM)-induced PF in mice [9]. Moreover, a small-molecule inhibitor J2 also inhibited radiation (IR)-induced PF (RIPF) in mice [8]. Regulation of EMT involves HSP27-mediated PF development [10], and NFkB activation via their direct interaction [8] is a suggested mechanism. Moreover, lung tissues of IPF patients have been shown to have increased expression of HSP27 and pHSP27 [11, 12].

Phosphorylation of HSP27 not only regulates its structural organization, but also affects its biological functions [13]. Recent studies have unveiled the distinct roles of phosphorylated HSP27 in cellular processes, but the detailed functions of the phosphorylated forms of HSP27 need to be further explored, especially in PF development.

Even though transcriptional regulation of *hsp27* is well recognized [14], few papers focus on degradation mechanisms of HSP27 protein. SMAD-specific E3 ubiquitin protein ligase 2 (Smurf2), which is a crucial part of the ubiquitin–proteasome pathway, was suggested to be involved in the degradation of HSP27 [15] without elucidation of a detailed mechanism.

miRNAs (miRs) are 18- to 25-nucleotide long noncoding RNAs that regulate pathophysiological functions via expression of target genes at the posttranscriptional level. Despite the evolutionary conservation of miRs, a single miR can target thousands of mRNAs, resulting in a lack of clarity about their true function or place in pathophysiologic events, including those that are central to the development of PF. Approximately 10% of miRs are significantly changed in human IPF lungs [16]. However, little is reported about the miRs involved or the targets of miR during development of PF.

Previously, we developed a mouse model simulating clinical stereotactic body radiotherapy (SBRT) and validated the induction of PF [17]. In addition, we identified molecular targets in PF development. HSP27 expression was increased during PF, and functional inhibition of HSP27 using a small molecule ameliorated PF. IkBα-NFkB signaling activation by direct interaction of IkBα with HSP27 is involved in the EMT process, which is tightly connected to the development of PF [8]. In this study, we further identified that inhibition of HSP27 degradation was involved in HSP27 accumulation during PF development. Transcriptional inhibition of Smurf2, which was mediated by induction of miR, resulted in inhibition of HSP27 degradation, especially degradation of phosphorylated HSP27, and finally promoted PF development.

## Methods

### Animal experiments

All procedures were approved by the Animal Care and Use Committees of Yonsei University Medical School (2015–0267) and were performed in accordance with the relevant guidelines. A single dose of 75 or 90 Gy was delivered using an X-RAD 320 platform (Precision x-ray, North Branford, CT) as described previously [17]. Lung tissues (n ≥ 3 per group) were collected at each time point after IR. The generation of HSP25 TG mice and establishment of the orthotopic lung tumor model were described previously [8]. Mice were administered i.p. J2 (15 mg/kg) on alternate days for indicated periods after 75 Gy IR.

C57BL/6 N (male, 6 weeks old) were purchased from Central Lab. Animal Inc. (Seoul, Korea) and housed in a pathogen-free and light-controlled room (12 h light and 12 h dark) with free access to food and water. Bleomycin (BLM) sulfate from Santacruz Biotechnology (Dallas, TX, USA) was used to induce fibrosis. The mice were anesthetized and treated with either saline (n = 3–4 mice) or BLM (n = 3–4 mice) (2.5 U/kg of body weight) in saline solution through an intratracheal injection. At 14 days post-BLM administration, the mice were sacrificed, and the lungs were removed for histological staining. All animal experiments were performed in accordance with protocols approved by the Institutional Animal Care and Use Committee of Ewha Womans University.

### Generation of HSP25 TG mice

HSP25 Mice was generated by Macrogen, Inc and mice were interbred and maintained in pathogen-free conditions at Macrogen, Inc (Seoul, Korea). All animal experiments were conducted in accordance with the Macrogen Institutional Animal Care and Use Committee approval. Briefly PMSG and hCG were used to treat C57BL/6 N female mice for superovulation. PMSG (7.5 IU) and hCG were IP injected at 48-h intervals (5 IU) into the female mice at 5–8 weeks. After hCG Injection, the female mice were mated with C57BL/6 N stud male mice. Next day, the vaginal plug was examined and female mice were sacrificed, to harvest the fertilized embryos. HSP25 DNA was co-microinjected into a single-cell embryo. Standard microinjection procedures were used to generate transgenic mice (Macrogen, Seoul, Korea). DNA 4 ng/µL was microinjected directly into the male pronucleus of zygote using micromanipulator and the microinjected embryos were incubated at 37 ℃ for 1–2 h. Fourteen to 16 one-cell stage embryos were transplanted surgically into oviducts of pseudo-pregnant recipient mice (ICR). After the birth of F0 generation, genotypic testing of tail-cut samples for the presence of the transgene was performed and confirmed by PCR analysis of their genomic DNA. PCR screening was carried out using phenol-extraction method.

### Human tissues analysis

The study of tissue specimens of 6 patients with RIPF was approved by Severance Hospital, Yonsei University. The tissue of each patient contained an irradiated fibrotic and a non-irradiated normal area. A human IPF tissue array (catalogue no. LC561) comprising 28 cases was purchased from US Biomax (Derwood, USA).

### Cell culture and treatment

The human normal lung epithelial cell line (L132) was obtained from the American Type Culture Collection (ATCC, Rockville, MD, USA) and cultured in RPMI (Gibco, Gaithersburg, MD, USA) supplemented with 10% fetal bovine serum (Gibco) in a 37 °C incubator with 5% CO_2_. Human primary pulmonary fibroblasts (HPFs) and human primary small airway epithelial cells (HSAEpCs) were obtained from PromoCell. All cells were used within nine passages. Cell lines were tested by BioMycoX Mycoplasma PCR Detection Kit (JCBIO Co., Ltd) to ensure that they were mycoplasma-free.

### siRNA and plasmids

Mammalian expression vectors such as p3xflag-myc-HSP27S15A/S78A/S82A and p3xflag-myc-HSP27S15D/S78D/S82D were prepared and pCMV5B-Flag-Smurf2-WT was from Addgene (plasmid #11746). siRNAs against Smurf2 (sc-41675) and a control siRNA (sc-37007) were purchased from Santa Cruz Biotechnology (Dallas, TX, USA). For transient transfection of siRNA or plasmids, L132 cells were plated and incubated for 24 h to reach 70% confluency. Cells were then transfected with the designated plasmids in each experiment using Lipofectamine 2000 (Invitrogen) according to the manufacturers protocol.

### Bioinformatic analyses

Total RNA from the mouse lung tissues was extracted using the Easy-SpinTM total RNA extraction kit according to the manufacturer’s instructions (iNtRON Biotechnology, Seoul, Republic of Korea). Isolated total RNA was amplified and labeled using the Low RNA Input Linear Amplification kit PLUS (Agilent Technologies) and hybridized to a microarray containing approximately 44,000 probes (~ 21,600 unique genes), in accordance with the manufacturer's instructions (Agilent Mouse whole genome 44 K, Agilent Technologies). The arrays were scanned using an Agilent DNA Microarray Scanner (Agilent Technologies).

GSE18800, GSE161322, GSE150910 and GSE24206, publicly available gene expression datasets used for therapeutic response rate analysis, were obtained from the National Center for Biotechnology Information (NCBI) gene expression omnibus database (GEO).

### RNA isolation, qRT-PCR

Total RNA was isolated from the sample using TRIzol® reagent (Qiazen, Valencia, CA, USA). RNA purity and concentration were measured with a Nanodrop. RNA was reverse transcribed using a ReverTra Ace® qPCR RT Kit (TOYOBO, kita-ku, Osaka, Japan) following the manufacturer's protocol. The mRNA expression was assessed by real-time PCR using SensiFAST sybr Hi-Rox Mix (Bioline USA Inc, Taunton, MA, USA) with CFX96 Touch™ Real-Time PCR Detection System (Biorad, USA), equipped at Ewha Drug Development Research Core Center. The 2-ΔΔCt method was used to analyze the relative changes in gene expression based on real-time quantitative PCR. Gapdh was used as an internal control gene. Primer sequences for qRT-PCR are listed in Additional file 1: Table S1.

### Tissue histology and immunohistochemical and immunofluorescence staining

Mice were euthanized and lung tissues were harvested and fixed in 10% (v/v) neutral buffered formalin before preparation of paraffin sections. Paraffin-embedded sections were deparaffinized and stained with hematoxylin and eosin (H&E), using a Masson's trichrome stain kit or Sirius red stain kit (Sigma-Aldrich) to detect collagen.

Before immunohistochemistry, deparaffinized sections were in antigen-retrieval buffer (Abcam) for 30 min and next incubated with 0.3% (v/v) hydrogen peroxide in methanol for 10 min. Sections were blocked in normal horse serum at 37 °C incubator for 1 h and immunostained overnight at 4 °C with primary antibodies. The target proteins were visualized using ABC and DAB kits (Vector Laboratories) and counterstained with hematoxylin.

For immunofluorescence staining, cell or sections were stained with primary antibodies and incubated with Alexa 488- labeled anti-mouse, Alexa 568- labeled anti-rabbit, Alexa 647- labeled anti-goat labeled secondary antibodies (1:1000; Thermofisher) and counterstained with 4,6-diamidino-2-phenylindole dihydrochloride (DAPI; 3 mmol/L). Images were obtained using a Zeiss microscope Apotome (Cal Zeiss), equipped at Ewha Drug Development Research Core Center. The detailed protocol and antibodies for immunohistochemical and immunofluorescence staining were provided in Additional file 1: Table S2.

### In situ proximity ligation assay (PLA)

PLA was performed to detect the interaction between AAA/DDD (Flag) and Smurf2. To visualize the bound antibody pairs, the Duolink Detection Kit (Duo92008) with PLA PLUS and MINUS probes for mouse and rabbit (Sigma-Aldrich) was used, according to the manufacturer’s instructions.

### Experiments of miRNA

The putative miR-15a, miR-128, miR-195 and miR-203a target sequences of Smurf2 mRNA were determined using TargetScan, miRDB and miRSystem. Total RNA was extracted from cells using Qizol Reagent (Qiagen, Valencia, CA, USA) according to the manufacturer’s instructions. To analyze miRNAs expression, cDNA was synthesized by a Mir-X miRNA First-Strand Synthesis Kit (Takara, Dalian, China). The quantitative real-time PCR (qRT-PCR) was performed using Mir-X miRNA (Takara, Dalian, China).

Expression levels of miRNAs in mouse tissues were quantified by quantitative polymerase chain reaction (miRCURY LNA microRNA PCR kit; Exiqon, Vedbaek, Denmark) according to the manufacturer’s protocol.

The miRNA inhibitors and mimics of each miRNAs were purchased from Genolution (Seoul, Korea). Upon reaching 60–70% confluence, the L132 cells were transfected with 100 nM of inhibitors or mimics of miRNAs using Lipofectamine RNAiMAX (Invitrogen, Carlsbad, CA, USA) according to the manufacturer’s instructions. The expression levels of miRNAs were quantified 24 h after transfection and the cells were used for a western blot and qPCR analysis.

### Antibodies

Twist (GeneTex); p50, p65, Hsp27, Smurf2, Ubiquitin, and β-actin (Santa Cruz Biotechnology); phospho-Hsp27 (Ser78), phospho-Hsp27 (Ser82), phospho-IkBα (Ser32/36), and Lamin A/C, Flag (Cell Signaling Technology); IkBα, IL6, IL1β, pro-SPC, and phospho-Hsp27 (Ser15) (Abcam); phospho-Hsp27 (Ser86) (Thermo Fisher Scientific); α-SMA and Flag (Sigma); and Alexa488-conjugated phalloidin (Invitrogen). The detailed antibodies for immunoblotting, immunohistochemical and immunofluorescence staining were provided in Additional file 1: Table S2.

### Inflammation score

The histology score was scored according to Gallet et al. in 2011, and a score of severity for inflammatory infiltrates was established, from 0 (no alteration) to 3 (severe alterations), using the visual scale presented in this journal. This visual scale is 0 grade = no inflammatory infiltrate; Grade 1 = Some inflammatory elements and mast cells; Grade 2 = Frequent inflammatory infiltrates; Grade 3 = Ubiquiary inflammatory infiltrates [18].

### Statistical analysis

Comparisons of all results were performed by t.test, one- or two-way ANOVA, and Newman-Keuls test where indicated. The difference was considered statistically significant at P ≤ 0.05, P ≤ 0.01, and P ≤ 0.001. All statistical analyses were performed using GraphPad Prism 8.

## Results

### Gradual increase of HSP25 protein with its phospho-forms during PF development

**Fig. 1:**
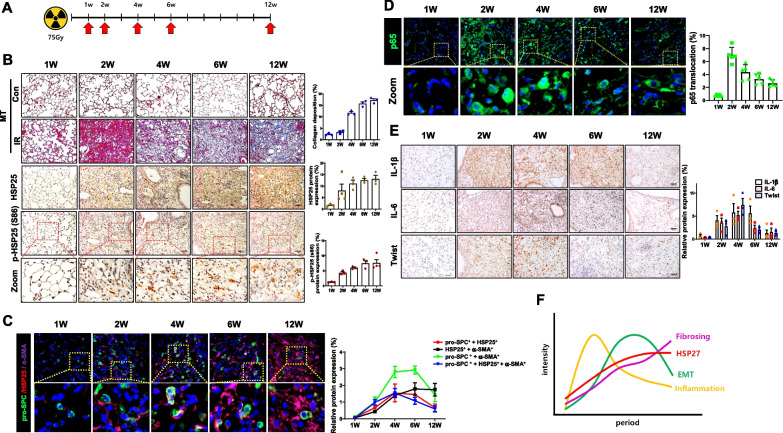
Expression of HSP25 protein during PF development. **A** Experimental scheme of focal exposure to high-dose radiation (75 Gy) to mice by period. **B** Representative images of mouse lung sections stained with Masson’s trichrome (top) at indicated times after focal 75 Gy irradiation (IR). Representative immunohistochemistry images of HSP25 and pHSP25 (S86) in mouse lung tissues (middle and bottom). Graphs show scores quantifying collagen deposition, and each protein -positive cells. Magnification, 200 × . Scale bar, 100 μm. **C** Pro-SPC (green) was used to identify type II AECs co-stained with HSP25 (red) and α-SMA (violet). Expression levels of HSP25 and α-SMA were upregulated in type II AECs of the irradiated lung tissue. Magnification, 400 × . Scale bar, 20 μm. Quantification of stained tissues was performed (n ≥ 3, mean ± SD). **D** Lung tissues were stained with anti-p65 antibody to investigate subcellular localization. Quantification of nuclear p65 level co-localized with DAPI was performed. Magnification, 400 × . Scale bar, 20 μm. (n ≥ 3, mean ± SD). **E** Representative immunohistochemical staining of Twist, IL-1β, and IL-6. Magnification, 200 × . Scale bar, 100 μm. Quantification of stained tissues was performed (n ≥ 3, mean ± SD). **F** Summary graph of RIPF in the mouse model used in this study. Inflammation represented by NFkB activation appeared initially, followed by EMT. Progression of fibrosis represented by α-SMA and expression of HSP27 continued to increase until the end of the experiment. The pattern remained the same

To explore the pathological assessment of PF, we initially established a PF mouse model using focal irradiation [17]. Using this model, mice were subjected to a single high-dose of 75 Gy IR, and lung tissues were collected on weeks 1, 2, 4, 6, and 12 to identify the pathological changes during lung fibrosis development (Fig. 1A). Lung sections were stained with H&E to determine the inflammation score and with Sirius red or Masson’s Trichrome (MT) to visualize the deposition of collagen. The inflammation score peaked 2 weeks after IR and gradually decreased as fibrosis developed. On the contrary, collagen deposition gradually increased until 12 weeks after IR, when extensive collagen deposition was observed and correlated with late-stage fibrosis. Also, HSP25 (HSP27 in humans and HSP25 in mice) protein expression in lung tissues gradually increased as lung fibrosis progressed based on immunohistochemical analysis. A previous study showed that the mRNA of HSP25 was not changed during RIPF. Posttranslational modification such as phosphorylation is suggested to be important for protein stability [19]. Serine (Ser)15 and Ser86 were the phosphorylation sites of mouse HSP25 and in the case of human HSP27, three phosphorylation sites at Ser15, Ser78, and Ser82 were identified [20–22]. pHSP25 at Ser86 was dramatically increased from 2 weeks after IR, with a peak increase at 6 weeks, similar to the pattern of total HSP25 expression. In addition, fluorescence staining of HSP25 was performed along with pHSP25 at Ser86. The double- stained HSP25 + /pHSP25 + area dramatically increased up to 12 weeks (Fig. 1B, and Additional file 2: Fig. S1A, and S2B). In contrast, the α-SMA expression was gradually increased and peaked at 12 weeks after IR, which was well correlated with the HSP25 expression pattern and fibrosis development. In the case of control mice, no collagen deposition was detected until the end of experiment. The co-localization of HSP25, pro-SPC, and α-SMA peaked at 4 weeks and then gradually decreased. The double-stained HSP25 + /pro-SPC + area dramatically increased up to 4 weeks and began to gradually decrease after 6 weeks. In contrast, the double-stained HSP25 + /α-SMA + area was gradually increased up to 12 weeks as fibrosis progressed (Fig. 1C and Additional file 2: Fig. S1C). Among the pro-SPC/α-SMA-positive cells, the proportion of HSP25-positive cells was high, especially in weeks 4 and 6, suggesting that most of the cells in which EMT (the transition of pro-SPC-positive cells to α-SMA-positive cells) occurs were HSP25-positive. A stable HSP27 knockdown cell line blocked the transition of pro-SPC + cells to α-SMA + cells (Additional file 2: Fig. S1D). When microarray analysis was performed using lung tissues after application of BLM in control B6 mice and HSP25 (mouse form of human HSP27) TG mice, expression of fibrosis-related genes increased, similar to that in irradiated mouse lungs (Additional file 2: Fig. S1E). We also examined NFkB activation during RIPF development and found that NFkB activation peaked at 2 weeks after IR (Fig. 1D). Immunohistochemistry of NFkB downstream molecules such as Twist, IL-1 β and IL-6 showed peak induction at 4 weeks after IR, which was the start point of the EMT (Fig. 1E). Summarizing the RIPF process used in this study as a schematic diagram, inflammation represented by NFkB activation was observed first and was followed by EMT. The expression of HSP27 and the progression of fibrosis represented by α-SMA showed the same pattern (Fig. 1F).

### Increased activation of NFkB by pHSP27 in fibrosis development

**Fig. 2:**
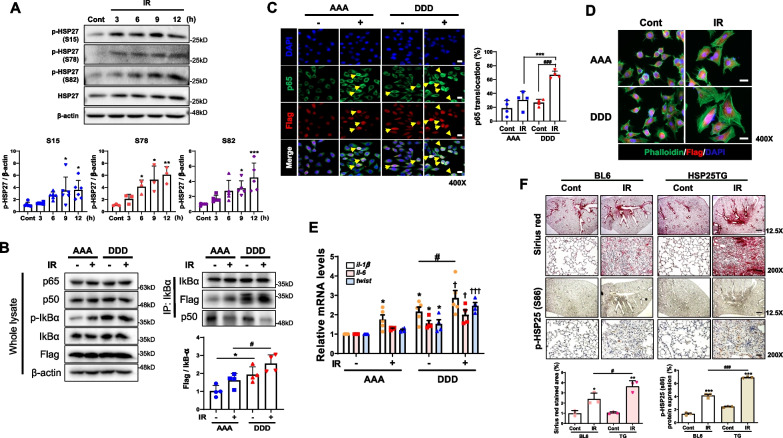
Increased expression of pHSP27 by IR**. A** Amounts of three phosphorylated HSP27 and total HSP27 proteins in the L132 cell line after 10 Gy IR at the indicated times by representative western blots and quantifications. ^*^P < 0.05, ^**^P < 0.01, ***P < 0.001 *vs*. Control **B** Immunoprecipitation and immunoblotting analyses using IkBα. L132 cells transfected with either AAA or DDD at 4 h after 10 Gy IR, displayed by representative western blots and quantifications. Ratio of each protein to β-actin in all western blot data (*n* ≥ 3). Biologically independent samples and results are representative of independent experiments. Data are expressed as mean ± SD. **C** Representative immunofluorescence image of p65 translocation in L132 cells transfected with either AAA or DDD at 4 h after 10 Gy IR (magnification, 400 ×). The graph shows the quantification of p65 and DAPI co-localization. **D** Representative immunofluorescence image of phalloidin (green) and DAPI (blue) in L132 cells transfected with either AAA or DDD (red). Magnification, 400 × **(E)** The mRNA levels of *il-1β*, *il-6*, and *twist* in L132 cells transfected with either AAA or DDD using qRT-PCR (n ≥ 3, mean ± SD). ^*^P < 0.05 *vs*. AAA-Control; ^*#*^*P* < 0.05 *vs*. DDD-Control; ^*†*^*P* < 0.05 and ^*†††*^*P* < 0.001 *vs*. AAA-IR. **F** Sirius red- and pHSP25 (Ser86)-stained lung Sections 6 weeks after focal 75 Gy irradiation (IR) in C57BL/6 N control and HSP25 TG mice. Graphs show scores quantifying collagen deposition and pHSP25-positive cells (n ≥ 3, mean ± SD). Magnification, 12.5 and 200 × . Scale bar, 100 μm. Subsequent statistical analysis was performed with unpaired one-way ANOVA with Newman-Keuls test for multiple comparisons

Similar to fibrotic lung tissues, in human lung epithelial L132 cells, IR increased pHSP27 at Ser15, Ser78, and Ser82 (Fig. 2A). In expansion of previous results that HSP27 activated NFkB by binding with IkBα, we investigate whether the phospho-forms of HSP27 affected HSP27 functions such as NFkB activation. These forms were a phospho-mimicking mutant involving replacement of three serine residues with aspartic acid (DDD) and a phospho-defective mutant with alanine (AAA) in place of three serine residues. Compared to the AAA form, the DDD form showed greater interaction with IkBα (IR induced more interaction between two molecules) even though the total levels of p65 and p50 were constant regardless of treatment (Fig. 2B). Moreover, DDD induced more frequent nuclear translocation of NFkB (p65) after IR exposure (Fig. 2C and Additional file 2: Fig. S2A). Decreased adhesion is a characteristic of cells with a mesenchymal phenotype. Staining with phalloidin (green) showed that immunofluorescent hair-like fibers protruding from cell surfaces into the collagen matrix assembled at the leading edge of the DDD-transfected cells after IR, whereas AAA reduced these protrusions (Fig. 2D). IR-induced mRNA expression of *il-1β, il-6,* and *twist* was greater in the DDD-transfected cells than in those with AAA (Fig. 2E), suggesting that IR-mediated pHSP27 induced greater activation of NFkB and EMT. When pHSP25 was examined in HSP25 TG mice after IR, higher expression of pHSP25 and more severe fibrosis were observed than those for irradiated lungs of control BL6 mice (Fig. 2F).

### Accumulation of pHSP27 by decreased expression of Smurf2 during fibrosis development

**Fig. 3:**
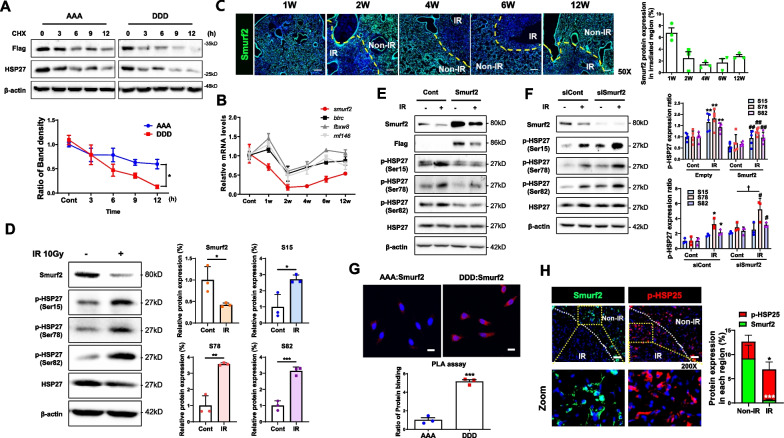
Inverse correlation of Smurf2 and pHSP27 during RIPF. **A** L132 cells were transfected with either non-phosphorylated alanine (HSP27S15A/S78A/S82A; AAA) plasmids or phosphomimetic aspartate (HSP27S15D/S78D/S82D; DDD) plasmids and treated with 10 μg/mL of cycloheximide (CHX) for various time periods. **B** Confirmation of ubiquitin-related genes by quantitative RT-PCR using lungs from individual mice. **C** Smurf2 was stained for immunofluorescence (green). The graph shows Smurf2 expression of a directly irradiated region. Magnification, 50 × . Scale bar, 200 μm. **D** Western blots using cell lysates 12 h after 10 Gy IR in L132 cells, displayed by representative blot and quantifications. **E** Lysates of L132 cell lines with an empty vector or Smurf2 WT (Smurf2) 24 h after 10 Gy IR, shown by representative blot and quantifications. **F** Western blots using cell lysates 24 h after 10 Gy IR in L132 cells with siControl or siSmurf2 transfection. Ratio of each protein to β-actin in all western blot data (*n* ≥ 3). Biologically independent samples and results are representative of independent experiments (D-F). Data are expressed as mean ± SD. ^*^P < 0.05, ^**^P < 0.01 and ^***^P < 0.001 *vs*. Control; ^*#*^*P* < 0.05 and ^*##*^*P* < 0.01 *vs*. Control-IR; ^*†*^*P* < 0.05 and ^*††*^*P* < 0.01 *vs*. treated and non-IR. **G** Proximity ligation assay (PLA) confirms endogenous AAA/DDD (Flag): Smurf2 interaction. Interactions with target proteins are indicated as red dots. Cell nuclei were counterstained with Hoechst (blue). Scale bar 10 μm. ^***^P < 0.001 *vs*. AAA (n ≥ 3, mean ± SD). **H** Immunofluorescence staining for Smurf2 (green) and pHSP25 (red) in RIPF mouse lungs at 6 weeks after 75 Gy IR. Magnification, 200 × , Scale bar, 50 μm. (n ≥ 3, mean ± SD)

To investigate whether the phospho-forms of HSP27 affected the stability of HSP27, DDD and AAA were transfected and treated with cycloheximide (CHX). Flag-tagged AAA showed a longer half-life than treated DDD, suggesting that phosphorylated HSP27 has a shorter half-life than unphosphorylated HSP27 (Fig. 3A). We hypothesized that degradation of phosphorylated HSP27 might be inhibited during PF. To confirm this, the expression patterns of several ubiquitin proteases from microarray data of PF tissues were examined and found that the expression of *btrc, fbxw8, rnf146*, and *smurf2* were altered. And then, we also observed the expression of these 4 genes in lung tissues of IPF patients using the NCBI GEO database (accession numbers GSE150910 and GSE24206). We confirmed that only the *smurf2* gene expression pattern was well correlated with the severity of IPF (Additional file 2: Fig. S2B and S2C). Indeed, compared to the other ubiquitin proteases, reduced mRNA and protein levels of *smurf2* were obviously observed in irradiated fibrotic lung tissues (Fig. 3B, C). Moreover, IR decreased the Smurf2 mRNA and protein levels and increased all three sites of pHSP27 in the L132 cell system (Fig. 3D and Additional file 2: Fig. S2D). To elucidate whether Smurf2 was directly involved in ubiquitin degradation of HSP27, proteasome inhibitor MG132 was applied to L132 cells, human primary fibroblast cells (HPFs) and small airway epithelial cells (HSAEpCs). The inhibition of ubiquitin degradation of all three pHSP27 forms, as well as that of total HSP27, was observed after MG132 treatment, and IR no longer increased the expression pHSP27 even though reduction of *smurf2* expression by IR remained (Additional file 2: Fig. S2E-G). Smurf2 overexpression increased the ubiquitin degradation of pHSP27, while siRNA of Smurf2 decreased it (Fig. 3E, F and Additional file 2: Fig. S2H). Smurf2 strongly interacted with DDD but not with AAA. Notably, this interaction was absent on treatment with IR (Additional file 2: Fig. S2I), which suggests that Smurf2 interacted with pHSP27 and induced ubiquitin degradation of pHSP27. Using a Proximity Ligation Assay (PLA), which can detect protein–protein binding in situ at a single-molecule resolution, endogenous interactions and localized protein-interaction complexes between DDD or AAA and Smurf2 were examined. A PLA for DDD: Smurf2 produced abundant red puncta in L132 cells, while that for AAA: Smurf2 produced only a few red puncta, suggesting that the DDD-Smurf2 interaction is stronger than the AAA-Smurf2 interaction (Fig. 3G). Immunofluorescence staining of lung tissues in a mouse RIPF model showed an inverse correlation between pHSP25 and Smurf2. The pHSP25 expression was dominantly induced in the irradiated fibrotic region; however, the expression of Smurf2 was observed in the non-IR region only (Fig. 3H).

**Fig. 4:**
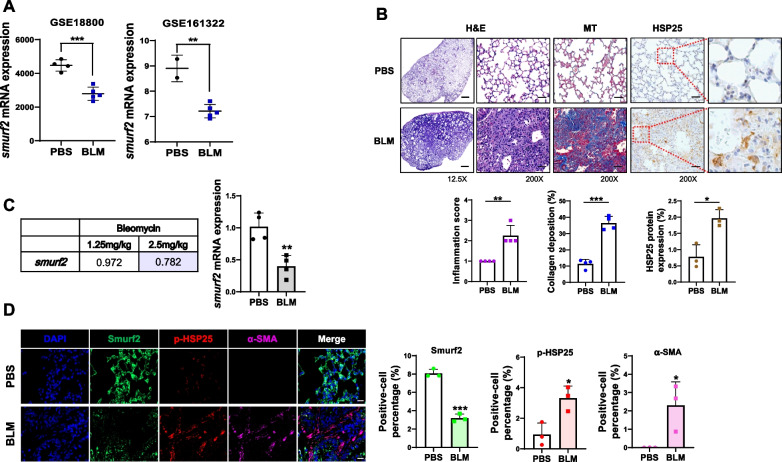
Inverse correlation between pHSP25 and Smurf2 in BLM-induced fibrosis models. **A** Analysis of smurf2 mRNA expression level in PBS control mice and lung tissues of BLM-induced lung fibrosis mice analyzed using the GSE18800 (left) and GSE161322 (right) datasets (mean ± SD). ^**^P < 0.01 and ^***^P < 0.001 *vs*. PBS control. **B** Representative images of mouse lung sections stained with H&E or Masson’s trichrome and HSP25 expression. Graphs show scores quantifying inflammation, collagen deposition, and HSP25-positive cells. Magnification, 12.5 × . Scale bar, 100 μm. 200 × . Scale bar, 100 μm. ^*^P < 0.05, ^**^P < 0.01 and ^***^P < 0.001 *vs*. PBS control. **C** Microarray data of smurf2 mRNA (left)**.** Confirmation of smurf2 mRNA by quantitative RT-PCR using lungs from individual mice (right). **D** Sections from control and BLM-induced lung fibrosis mice tissues were stained for Smurf2 (green), pHSP25 (Red), and α-SMA (violet). Representative images of each specimen are shown. The data from 3 specimens are quantitated. The graph shows the intensity of co-localization of Smurf2 (green) and pHSP25 (red) or pHSP25 (red) and α-SMA (violet) in fibrotic areas from 30 independent views in each sample. Magnification, 400 × . Scale bar, 20 μm. ^*^P < 0.05 and ^***^P < 0.001 *vs*. PBS control

mRNA of *smurf2* was also decreased in BLM-induced fibrotic lung models based on the NCBI GEO database (accession number GSE18800 and GSE161322) (Fig. 4A). To explore the relationship of *smurf2* and pHSP25, we established a PF mouse model by BLM. BLM-induced fibrotic lung tissues also showed increased HSP25 (Fig. 4B). Using cDNA microarray data and qPCR, we determined decreased expression of *smurf2*, similar to that of RIPF (Fig. 4C). Also, an inverse correlation between Smurf2 and pHSP25 was observed in fibrotic tissues (α-SMA expression) (Fig. 4D). Therefore, Smurf2 downregulation and increased pHSP25 are common phenomena during PF.

### Involvement of miRNA15b for downregulation of Smurf2

**Fig. 5:**
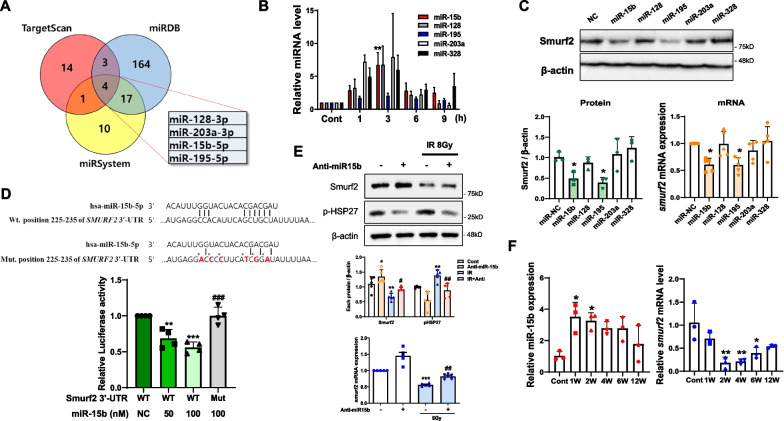
Involvement of miR-15b in downregulation of Smurf2. **A** Online prediction databases (TargetScan, miRDB, and miRSystem) were used to predict miRNAs targeting Smurf2. **B** miRNA expression measured by qPCR at each time point after 10 Gy IR. ^**^P < 0.01 *vs.* control. **C** Cell lysates 24 h after transfection to mimic RNAs (100 nM). Analysis was performed by western blot and qRT-PCR; *gapdh* mRNA was used for normalization. ^*^P < 0.05 *vs*. miR-NC. **D** Putative miR-15b binding site in the human Smurf2 3’-UTR and luciferase constructs with the wild-type (WT) and mutant (Mut) miR-15b target sequences. The red colors indicate the mutant sequences of the 3’-UTR (upper). L132 cells were co-transfected with 50 and 100 nM of the miR-15b together with the pmirGLO dual-luciferase vectors containing the wild type or mutant 3’-UTR of Smurf2. Data are represented as the mean ratios of Renilla to Firefly luciferase activity and are normalized relative to the negative control (bottom). ^**^P < 0.01 and ^***^P < 0.001 *vs*. WT-NC; ^*###*^*P* < 0.001 *vs*. WT-100 nM. **E** Cell lysates 9 h after 8 Gy IR with or without anti-miR-15b (50 nM). Analysis was performed by western blot and qRT-PCR; *gapdh* mRNA was used for normalization. Ratio of each protein to β-actin in all western blot data (*n* ≥ 3, mean ± SD). Subsequent statistical analysis was performed with one-way ANOVA with Newman-Keuls test for multiple comparisons. ^*^P < 0.05, ^**^P < 0.01, and ^***^P < 0.001 *vs*. control; ^*#*^*P* < 0.05 and ^*##*^*P* < 0.01 *vs*. IR only. **F** Periodic expression was confirmed by qRT-PCR using lungs from individual mice. The miR-15b expression was normalized to that of U6. The *smurf2* expression was normalized to that of *gapdh* (n = 3, mean ± SD). ^*^P < 0.05 and ^**^P < 0.01 *vs*. control

Smurf2 was transcriptionally reduced by IR, and several studies have indicated the regulation of *smurf2* mRNA by miRs [23–25]. To investigate the functional relation between Smurf2 and miRs in PF, we searched candidate target miRs of Smurf2. Using three commonly used prediction databases, TargetScan, miRDB, and miRSystem, we found that miR-15b, -128, -195, and -203a could target the 3'‑UTR of Smurf2 (Fig. 5A). Indeed, when qRT-PCR for these four miRs was performed after 10 Gy IR in L132 cells, their expression was increased, except for that of miR-195 (Fig. 5B). miR-328 has been reported to be increased by IR [26] and was used as a positive control here. To elucidate the relationships between Smurf2 and these miRs, a mimic of each miR was transfected, and Smurf2 expression was examined. The mimic of miR-15b, but not those of miR-128 and -203a, inhibited Smurf2 expression (Fig. 5C). So, we selected miR-15b as a Smurf2 regulator under radiation. To further confirm whether miR-15b directly targets Smurf2, we conducted dual luciferase reporter assay using the Smurf2 3’-UTR wild type (WT) or mutated sequence (Mut). Dual luciferase reporter assay showed that miR-15b decreased the luciferase activity of WT Smurf2. However, the Mut Smurf2 did not show any changes of luciferase activity (Fig. 5D). Indeed, anti-miR-15b treatment restored the Smurf2 expression even after IR (both protein and mRNA levels) and finally resulted in the inhibition of pHSP27 expression (Fig. 5E). We also examined the increased expression of miR-15b by qRT-PCR at very early time points (1 and 2 weeks) in the RIPF model. In addition, *smurf2* mRNA began to decrease from 1 week and showed a maximum decrease at 2 and 4 weeks (Fig. 5F). These findings indicate that miR-15b directly regulate the Smurf2 gene expression through post-transcriptional repression.

### Inverse correlation between pHSP27 and Smurf2 in mouse fibrotic lung tissues, irradiated orthotropic lung tumor, and human fibrotic lung tissues

To evaluate these phenomena in lung tumor model after RT, orthotropic lung tumors using LLC1 mouse lung cancer cells were established, and the whole left lung of each mouse was irradiated with 90 Gy IR. The irradiated orthotropic lung tumor model is a model simulating lung cancer patient with RT. IR dramatically decreased the detectable residual tumors in mice, while it greatly increased collagen deposition and HSP25 expression in the irradiated tissues (Additional file 2: Fig. S3A). Immunofluorescence of Smurf2 and pHSP25 showed an inverse correlation between these two proteins, similar to the results obtained with the RIPF model (Additional file 2: Fig. S3B). We also examined pHSP25 and Smurf2 in tumor tissues and observed an inverse correlation between them (Additional file 2: Fig. S3C), similar to that observed in fibrotic lungs.

**Fig. 6:**
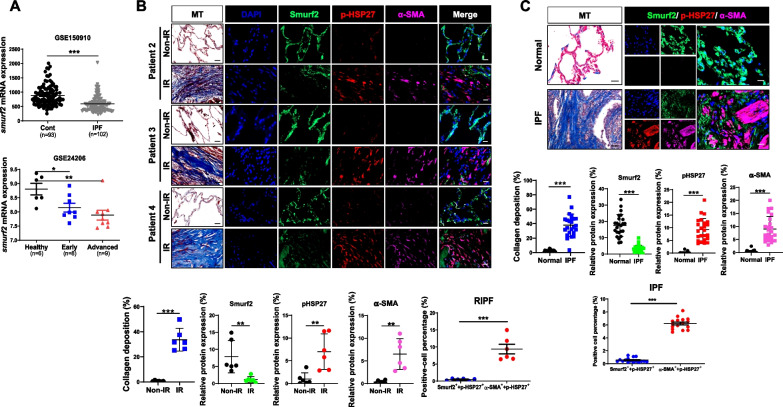
Inverse correlation between pHSP27 and Smurf2 in lung tissues of RIPF and IPF patients. **A** Analysis of smurf2 mRNA expression level in healthy human lung tissues (n = 98) and lung tissues of IPF patients (n = 102), analyzed using the GSE150910 dataset (upper). Analysis of relative mRNA of smurf2 level in healthy human lung tissues (n = 6) and in lung tissues of patients with early (n = 8) and advanced (n = 9) IPF using GSE24206 data (bottom) (mean ± SD). ^*^P < 0.05, ^**^P < 0.01, and ^***^P < 0.001 *vs*. control. **B** Sections from human RIPF tissues were stained for Smurf2 (green), pHSP27 (red), and α-SMA (violet). Representative images of each specimen are shown. The data from 6 specimens are quantitated. The graph shows the intensity of co-localization of Smurf2 (green) and pHSP27 (red) or pHSP27 (red) and α-SMA (violet) in fibrotic areas from 30 independent views in each sample. Magnification, 400 × . Scale bar, 20 μm. **C** Sections from human control and IPF tissues were stained for Smurf2 (green), pHSP27 (Red), and α-SMA (violet). Representative images of each specimen are shown. The data from 16 specimens are quantitated. The graph shows the intensity of co-localization of Smurf2 (green) and pHSP27 (red) or pHSP27 (red) and α-SMA (violet) in fibrotic areas from 30 independent views in each sample (n = 24). Magnification, 400 × . Scale bar, 20 μm. ^**^P < 0.01, and ^***^P < 0.001 *vs*. Non-IR or normal

Next, we observed these phenomena in humans. Gene expression of *smurf2* using a cohort of IPF tissues (102 IPF patients and 93 normal lung samples/8 early IPF patients, 9 advanced IPF patients, and 6 healthy lung samples) available from the NCBI GEO database (accession number GSE150910 and GSE24206) indicated that the *smurf2* gene level was significantly downregulated in the tissues of IPF patients. In addition, the gene expression patterns of *smurf2* were well correlated with the severity of IPF (Fig. 6A). When we used tissues of RIPF patients who had undergone surgery following RT for lung adenocarcinoma (6 patients), increased collagen deposition was observed in the irradiated area compared to the unirradiated area (Additional file 2: Fig. S4A). Immunofluorescence data for pHSP27, Smurf2, and α-SMA suggest that pHSP27 expression was co-localized in an α-SMA-positive area. However, Smurf2 was expressed in a non-fibrotic area with lower expression of pHSP27 (Fig. 6B and Additional file 2: Fig. S4B). The clinicopathological characteristics of the patients are summarized in Additional file 1: Table S3. Similar to lung tissues of RIPF patients, an examination of the lung tissues of IPF patients (n = 24) indicated an inverse correlation between pHSP27 and Smurf2 (Fig. 6C and Additional file 2: Fig. S5A-C). The pHSP27 expression was co-localized with α-SMA-positive areas, while Smurf2 was expressed in non-fibrotic areas with lower expression of pHSP27.

### Targeting EMT using a HSP27 cross linker for inhibition of RIPF

**Fig. 7:**
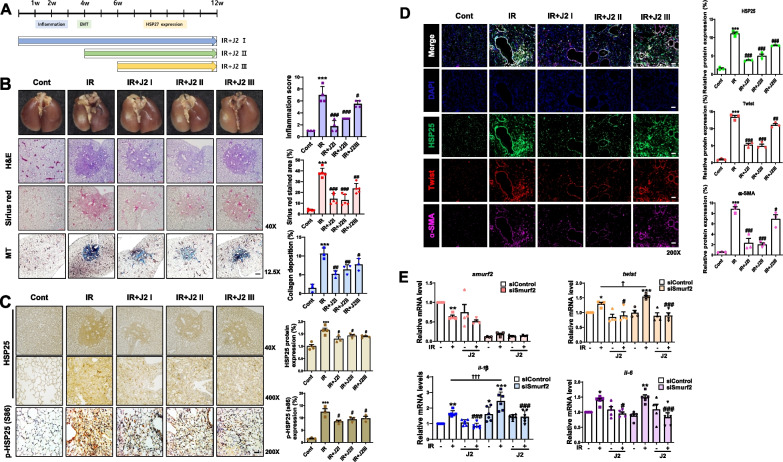
Targeting the EMT stage for inhibition of PF by HSP27 inhibitor. **A** Illustration of the differences in HSP27 cross-linker administration periods. All mice were intraperitoneally (i.p.) administrated J2 (15 mg/kg); IR + J2 I group: J2 treatment until 12 weeks after 75 Gy IR exposure; IR + J2 II group: J2 treatment from 4 to 12 weeks after IR; IR + J2 III group: J2 treatment from 6 to 12 weeks after IR. On the 12th week, the lung tissues of mice from each group were harvested. **B** Lungs were photographed after complete fixation (top) and stained with H&E (2nd from the top), Sirius red (2nd from the bottom), or Masson’s trichrome (bottom). The graph shows quantification of the stained region. n ≥ 3, mean ± SD; ^***^P < 0.001 *vs*. control; ^*#*^*P* < 0.05, ^*##*^*P* < 0.01 and ^*###*^*P* < 0.001 *vs*. IR only. **C** Immunohistochemistry of HSP25 (top and middle; 40 and 400 ×) and pHSP25 (Ser 86) (bottom; 200 ×) in mouse lung tissues. The graph shows the quantification of positive cells. n ≥ 3, mean ± SD; ^***^P < 0.001 *vs*. control; ^#^P < 0.05 *vs*. IR only. **D** HSP27 (green) was co-stained with Twist (red) and α-SMA (violet). Magnification, 200 × . Scale bar, 20 μm, (n ≥ 3). ^***^P < 0.001 *vs*. control; ^*#*^*P* < 0.05, ^*##*^*P* < 0.01, and ^*###*^*P* < 0.001 *vs*. IR only. **E** qRT-PCR of L132 cells transfected with either siControl or siSmurf2. Cell lysates 12 h after 10 Gy IR with or without J2 (10 μM). *gapdh* mRNA was used for normalization. (n ≥ 4, mean ± SD) ^*^P < 0.05, ^**^P < 0.01, and ^***^P < 0.001 *vs*. control; ^*#*^*P* < 0.05 and ^*###*^*P* < 0.001 *vs.* IR alone; ^*†*^*P* < 0.05 and ^*†††*^*P* < 0.001 *vs*. siControl-IR

It has been reported that Smurf2 appears to act as an oncogene, promoting tumor development through stabilization of KRAS and EGFR [27]. Therefore, to design a more desirable approach for PF treatment, inhibiting HSP27 is preferable to activating Smurf2. For this purpose, we inhibited HSP25 using functional inhibitor J2 by altered cross linking of HSP25 [28] at the different stages of PF to elucidate the mechanisms related to the timing of HSP25 inhibition: whole period treatment (for 12 weeks, IR + J2I), treatment starting at the EMT stage (after 4 weeks of IR and total 8 weeks of treatment, IR + J2II), and treatment starting at the advanced fibrosis stage (after 6 weeks of IR and total 6 weeks of treatment, IR + J2III) (Fig. 7A). Both the inflammation score and collagen deposition were significantly inhibited in all the groups; however, inhibition of inflammation and collagen deposition was weaker in IR-J2III compared to IR + J2I or IR + J2II (Fig. 7B), even though expression of HSP25 and pHSP25 was similarly inhibited in all 3 groups (Fig. 7C). With co-staining of α-SMA and HSP25, IR + J2I and IR + J2II showed dramatic inhibition of the co-expression, while IR + J2III showed slight inhibition (Fig. 7D). This suggests that HSP25 inhibition at the EMT stage is critical for inhibition of RIPF. The expression of pHSP25 was greater in PF tissues of TG mice than in those of BL6 control mice. J2 effectively inhibited the inflammation, deposition of collagen, and pHSP25 (Additional file 2: Fig. S6A and S6B). Increased transition from pro-SPC + , HSP25 + cells to α-SMA + , HSP25 + cells in PF was also inhibited (Additional file 2: Fig. S6C). Moreover, J2 induced altered cross-linking activity of both AAA and DDD (Additional file 2: Fig. S7A), suggesting that J2 induced cross linking activity of HSP27 regardless of its phosphorylation status, and that IR did not affect altered cross-linking activity of J2 (Additional file 2: Fig. S7B). When siRNA of Smurf2 was applied to L132 cells, J2 effectively inhibited IR-mediated NFkB activation, which was detected by downstream effects of NFkB activation such as those to mRNA of *twist, il-1β,* and *il6* (Fig. 7E). High-dose IR was used in this RIPF model to simulate clinical SBRT, and fibrosis was rapidly induced. To simulate the slow fibrosis process, a lower IR dose of 20 Gy was applied, and J2 was administered for 12 months, at which peak fibrosis was induced (Additional file 2: Fig. S8A). In this model, J2 efficiently inhibited inflammation scores and collagen deposition (Additional file 2: Fig. S8B). Similar to the high-dose IR model, J2 inhibited the expression of pHSP25 and total HSP25 (Additional file 2: Fig. S8C). J2 did not induce toxicity in the high- or low-dose RIPF model based on body weight (Additional file 2: Fig. S8D).

## Discussion

This study showed that miR-15b-mediated Smurf2 downregulation is involved in HSP27 stabilization, especially that of pHSP27, which promoted PF. Because the mRNA of HSP27 did not change in PF, we hypothesized that posttranslational modification of HSP27 was involved in the development of PF. Indeed, the expression of pHSP27 increased during PF; in addition, its pattern was similar to that of total HSP27 expression. pHSP27 showed a shorter half-life, suggesting that its protein degradation pathways were involved in PF development, and that inhibition of pHSP27 degradation affected PF.

There are only few studies on degradation mechanisms of HSP27 protein. Smurf2 is a crucial part of the ubiquitin–proteasome pathway that regulates cellular signal transduction via ubiquitin-dependent degradation of some substrates and receptors. It has been suggested to be involved in ubiquitylation-dependent degradation of HSP27 through interaction [15]. Our microarray data obtained using the lung tissues of a PF model revealed a significantly positive correlation with the downregulated *smurf2* gene. Smurf2 knockdown inhibited the ubiquitin degradation of HSP27 by their interaction, which was potentiated in pHSP27. Smurf2 expression was more dominant in non-fibrotic areas; however, pHSP27 and α-SMA were highly expressed in fibrotic areas, suggesting an inverse correlation between Smurf2 and pHSP27 during PF. Moreover, Smurf2 has a significant role in ubiquitin‐mediated proteasomal degradation of TGF-β signaling components such as SMAD1, SMAD2, and TGFβRI [29–32], as with fibrosis development.

An analysis using three commonly used prediction databases and further detailed experiments identified miR-15b for inhibition of *smurf2* transcription. Indeed, the miR-15b expression increased with the progression of PF. Inverse correlation between Smurf2 and pHSP27 was observed in fibrotic tissues in orthotropic lung tumors after irradiation, simulating model for RT. Moreover, tissues of patients with RIPF and IPF indicated an inverse correlation between Smurf2 and pHSP27 in fibrotic regions, suggesting their physiological relevance in human PF.

We have previously demonstrated the role of HSP27 in the development of PF and proposed HSP27 as a possible therapeutic target. Increased HSP27 aggravated NFkB signaling pathways to increase EMT, and nontoxic pharmacological HSP27 inhibitors such as J2 could be used as inhibitors of PF [8]. J2 efficiently inhibited PF when treatment was started at the inflammation or EMT stage. We also examined the effects of delayed J2 treatment at the late fibrosis stage, i.e., 6 weeks after IR injury for up to an additional 6 weeks. Only slight inhibition was seen, suggesting that HSP27 inhibition at the EMT stage is critical for inhibition of PF. The reason for the dominant J2 effects upon treatment at the EMT stage was inhibition of HSP27-mediated NFkB activation. Thus, inhibition of NFkB activation by J2 was considered a key step in the pro-fibrotic process including EMT. Because EMT is suggested to be essential for development of PF [33] and HSP27 is known to be involved in various mechanisms of EMT in fibrosis [7–10], inhibition of EMT by HSP27 inhibition is expected to provide a good treatment option for PF, which currently has few suitable treatment options. Another clinical implication of HSP27 inhibitors is that J2 efficiently inhibited HSP27 function even in Smurf2-downregulated fibrotic tissues, which is similar status with RT. Inhibition of Smurf2 expression by miR-15b began at the initial stage of inflammation, where accumulation of pHSP27 was also observed and might be another reason why HSP27 inhibitors are used before progression to advanced fibrosis. This knowledge of treatment with HSP27 inhibitors could provide important clues for clinical application.

Another important finding in this study is that the HSP27 inhibition strategy can be applicable to IPF and RIPF. Although there are several controversies about the similarity and differences of pathological mechanisms between IPF and RIPF [34, 35], HSP27 expression is well recognized to be increased in lung tissues of IPF [11, 12] and animal models of BLM-induced lung fibrosis [9], as well as an RIPF animal model and RIPF patients in this study. Moreover, our results showed Smurf2 downregulation in lung tissues of IPF patients with relevance to clinical severity, as well as in a BLM-induced lung fibrosis animal model. Even though currently approved therapies for IPF such as pirfenidone and nintedanib are clinically available, there are some limitations in side effects and effectiveness. Therefore, an alternate strategy to address the unmet therapeutic need of lung fibrosis is necessary, and the development and clinical application of novel HSP27 inhibitors will provide an additional therapeutic option for overcoming IPF.

## Conclusions

**Fig. 8:**
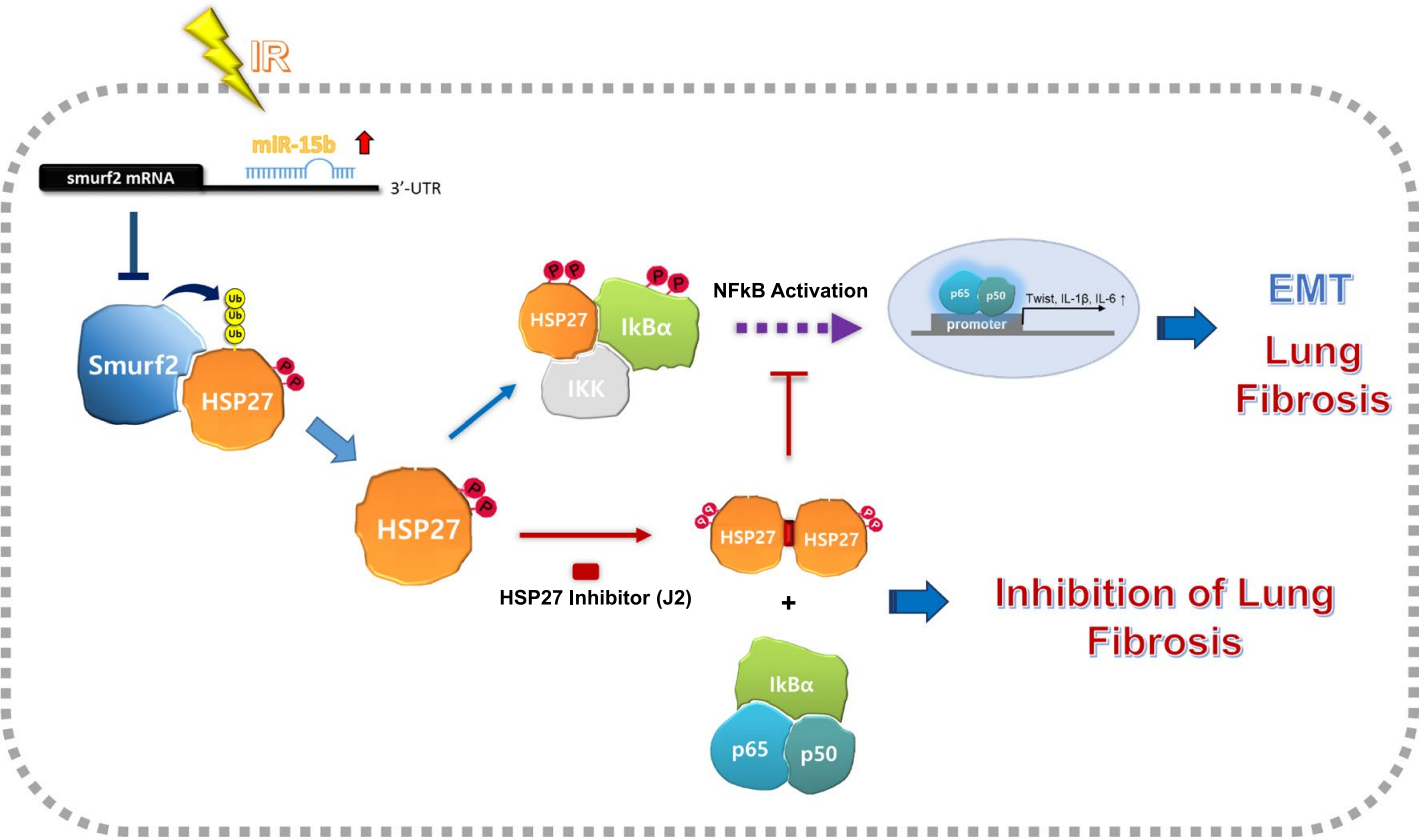
Schematic illustration of molecular mechanism of PF via the miRNA-15b/Smurf2/HSP27 axis. The miR-15b transcriptionally inhibited Smurf2 expression. Decreased expression of Smurf2 by miR-15b is involved in the increased protein stability of HSP27, especially that of pHSP27. Accumulation of pHSP27 exacerbated PF through NFkB-mediated EMT progression. Inhibition of EMT by HSP27 inhibitors at the EMT stage has potential for clinical application for overcoming PF

In conclusion, the increase in expression of miR-15b transcriptionally inhibited Smurf2 expression. As a result, degradation of pHSP27 was suppressed, and accumulation of pHSP27 exacerbated PF. This is because HSP27 affected the fibrosis caused by EMT progression, which was mediated by NFkB activation. To our knowledge, we are the first to demonstrate that the microRNA-Smurf2-HSP27 axis promotes PF, and inhibition of lung fibrosis can be accelerated by a pharmacological intervention that targets the EMT. We also provide the mechanistic insights of HSP27-mediated EMT during lung fibrosis development and suggest inhibition of HSP27 as a good clinical application for overcoming PF (Fig. 8).

## Supplementary Information

**Additional file 1: Table S1.** Primer sequence. **Table S2.** Antibody information.

**Additional file 2: Fig. S1.** Involvement of HSP27 in IR-induced EMT. **Fig. S2.** Decrease expression of Smurf2 by phosphorylated HSP27. **Fig. S3.** Inverse correlation between pHSP25 and Smurf2 in irradiated orthotropic lung tumor models. **Fig. S4.** Inverse correlation between pHSP27 and Smurf2 in RIPF patient tissues. **Fig. S5.** Inverse correlation between pHSP27 and Smurf2 in IPF patient tissues. **Fig. S6.** HSP25 cross-linker J2 inhibited IR-induced EMT and fibrosis development in mice. **Fig. S7.** J2 induced cross-linking activity of HSP27 as well as pHSP27. **Fig. S8. **Nontoxic pharmacological HSP25 inhibitor, J2 as an inhibitor of RIPF.

## Data Availability

Data supporting the present study are available from the corresponding author upon reasonable request.

## Abbreviations

BLM: Bleomycin
*btrc*: Beta-transducin repeat containing E3 ubiquitin protein ligase
CHX: Cyclohexamide
ECM: Extracellular matrix
EGFR: Epidermal growth factor receptor
EMT: Epithelial to mesenchymal transition
*fbxw8*: F-box and WD repeat domain containing 8
GEO: Gene expression omnibus
HPFs: Human primary pulmonary fibroblasts
HSAEpCs: Human primary small airway epithelial
HSP27: Heat shock protein 27
IL-1β: Interleukin-1β
IL-6: Interleukin-6
IPF: Idiopathic pulmonary fibrosis
IR: Radiation
KRAS: Kirsten rat sarcoma virus
LLC1: Lewis lung carcinoma cells
L132: Human normal lung epithelial cell line
miRs: Micro RNAs
PF: Pulmonary fibrosis
PLA: Proximity ligation assay
RIPF: Radiation-induced PF
*rnf146*: Ring finger protein 146
RT: Radiotherapy
SBRT: Stereotactic body radiotherapy
SMAD1: SMAD family member 1
SMAD2: SMAD family member 2
pro-SPC: Prosurfactant protein C
Smurf2: SMAD-specific E3 ubiquitin protein ligase 2
TGF-β: Transforming growth factor-β
TGFβRI: Transforming growth factor- β receptor 1
α-SMA: α-Smooth muscle actin
