# Supplementary material for: The miR-15b-Smurf2-HSP27 axis promotes pulmonary fibrosis
Source: J Biomed Sci. 2023 Jan 7;30:2. doi: 10.1186/s12929-023-00896-5 (PMC9824921; doi:10.1186/s12929-023-00896-5)
Supplement: Supplementary file 1 — Additional file 1: Table S1. Primer sequence. Table S2. Antibody information. [file 12929_2023_896_MOESM1_ESM.docx]

**Table S1. Primer sequence**

| **Gene** | **Species** | **Sequence** |
| --- | --- | --- |
| *twist* | human | Forward: GTC CGC AGT CTT ACG AGG AGC |
|  |  | Reverse: GCT TGA GGG TCT GAA TCT TGC T |
| *il-1β* | human | Forward: TGA GCT CGC CAG TGA AAT GA |
|  |  | Reverse: AAC ACG CAG GAC AGG TAC AG |
| *il-6* | human | Forword: TTC GGT CCA GTT GCC TTC TC |
|  |  | Reverse: CAG CTC TGG CTT GTT CCT CA |
| *smurf2* | human | Forward: TTG GCT CTG CAG AAA GGA TT |
|  |  | Reverse: CCA CAG CTT TCC AGA ACC AT |
| *gapdh* | human | Forward: TGT AGT TGA GGT CAA TGA AGG G |
|  |  | Reverse: ACA TCG CTC AGA CAC CAT G |
| *btrc* | mouse | Forward: TGC TCT ATG CCC AGG TCT CT |
|  |  | Reverse: TCC TAG GGG GTT CGC CAT TA |
| *fbxw8* | mouse | Forward: CCT GCC ACA TGT CTG GAT GT |
|  |  | Reverse: CTT ATC GCT GCG GAG GTC AT |
| *rnf146* | mouse | Forward: GGG CCG GAC CGA GAA AG |
|  |  | Reverse: TGG TGA CAA CAA GGT TGG CT |
| *smurf2* | mouse | Forward: CGA GGA AGC AGA AGT GGG AG |
|  |  | Reverse: TGC ACA GAG TAC TGT CAG GC |
| *gapdh* | mouse | Forward: ACT GTG GTC ATG AGC CCT TC |
|  |  | Reverse: GGG TGT GAA CCA CGA GAA AT |
| miR-15b | human | Forward: GGGGTAGCAGCACATCATG |
|  |  | Reverse: GTGCGTGTCGTGGAGTCG |
| miR-128 | human | Forward: GGT CAC AGT GAA CCG GTC |
|  |  | Reverse: GTG CAG GGT CC G AGG T |
| miR-195 | human | Forward: CGC AGC ACA GAA ATA TTG GC |
|  |  | Reverse: CTC AAC TGG TGT CGT GGA GTC |
| miR-203a | human | Forward: CCG GTG AAA TGT TTA GGA CCA CTA G |
|  |  | Reverse: GCC GCG TGA AAT GTT TAG G |
| miR-328 | human | Forward: GCC GAG CTG GCC CTC UCT GC |
|  |  | Reverse: CTC AAC TGG TGT CGT GGA |
| U6 | human | Forward: CTC GCT TCG GCA GCA CA |
|  |  | Reverse: AAC GCT TCA CGA ATT TGC GT |

**Table S2. Antibody information**

| **Antigen** | **Host** | **Supplier** | **Product number** |
| --- | --- | --- | --- |
| HSP27 | Mouse | Santa Cruz Biotechnology | Sc-13132 |
| Twist | Rabbit | GeneTex | GTX127310 |
| IL-1β | Rabbit | Abcam | Ab9722 |
| IL-6 | Rabbit | Abcam | Ab6672 |
| Pro-SPC | Rabbit | Abcam | Ab3786 |
| α-SMA | Goat | Novus biothechnology | NB300-978 |
| Smurf2 | Mouse | Santa Cruz Biotechnology | Sc-393848 |
| p-HSP27 (S15) | Rabbit | Biorbyt | **orb99259** |
| p-HSP27 (S78) | Rabbit | Cell signaling | 2405s |
| p-HSP27 (S82) | Rabbit | Cell signaling | 2401s |
| p-HSP25 (S86) | Rabbit | Invitrogen | 44-536G |
| Flag | Mouse | Sigma | F3165 |
| Flag | Rabbit | Cell signaling | 14793s |
| β-actin | Mouse | Santa Cruz Biotechnology | Sc-47778 |
| p65 | Mouse | Santa Cruz Biotechnology | Sc-8008 |
| p50 | Mouse | Santa Cruz Biotechnology | Sc-8414 |
| p-IkB | Rabbit | Cell signaling | 9246s |
| IkB | Mouse | Abcam | Ab32518 |
| Ubiquitin | Mouse | Santa Cruz Biotechnology | Sc-8017 |
| Alexa Fluor 488-phalloidin |  | Invitrogen | A-12379 |
| Alexa 488- labeled anti- mouse |  | Invitrogen | A-28175 |
| Alexa 568- labeled anti-rabbit |  | Invitrogen | A-11036 |
| Alexa 647- labeled anti- goat |  | Invitrogen | A-32849 |

**Table S3. The clinicopathologic characteristics of lung cancer patients**

| **Patient** | **Sex** | **Age** | **Pathology** | **Radiotherapy Dose** | **Radiotherapy Period** | **Radiotherapy to Surgery interval** | **Concurrent chemotherapy** | **Initial stage** | **Postop Stage** |
| --- | --- | --- | --- | --- | --- | --- | --- | --- | --- |
| **No.** |  |  |  |  |  |  | **(before RT/after RT)** |  |  |
| 1 | M | 58 | Adenocarcinoma | 66 Gy/33fx | 53 days | 62 days | Docetaxel/Cisplatin | icT2N3M0 | ypT1N0M0 |
| 2 | M | 69 | Squamous cell carcinoma | 50 Gy/25 fx | 34 days | 40 days | Docetaxel/Cisplatin | icT2N2M0 | ypT1N2M0 |
| 3 | M | 57 | Adenocarcinoma | 54 Gy in 30 fractions | 47 days | 49 days | Docetaxel/Cisplatin | icT3N2M0 | ypT0N0M0 |
| 4 | M | 73 | Adenocarcinoma | 53.4 Gy/29fx | 49 days | 142 days | Taxotere/Cisplatin | icT3N2M0 | ypT1N2M0 |
| 5 | M | 46 | Adenocarcinoma | 54 Gy in 27 fractions | 43 days | 46 days | Taxotere/Cisplatin | icT3N3M0 | ypT0N0M0 |
| 6 | F | 58 | Adenocarcinoma | 50 Gy in 25 fractions | 38 days | 61 days | Taxotere/Cisplatin | icT2N2M0 | ypT2N0M0 |
