# Supplementary material for: The miR-15b-Smurf2-HSP27 axis promotes pulmonary fibrosis
Source: J Biomed Sci. 2023 Jan 7;30:2. doi: 10.1186/s12929-023-00896-5 (PMC9824921; doi:10.1186/s12929-023-00896-5)
Supplement: Supplementary file 2 — Additional file 2: Fig. S1. Involvement of HSP27 in IR-induced EMT. Fig. S2. Decrease expression of Smurf2 by phosphorylated HSP27. Fig. S3. Inverse correlation between pHSP25 and Smurf2 in irradiated orthotropic lung tumor models. Fig. S4. Inverse correlation between pHSP27 and Smurf2 in RIPF patient tissues. Fig. S5. Inverse correlation between pHSP27 and Smurf2 in IPF patient tissues. Fig. S6. HSP25 cross-linker J2 inhibited IR-induced EMT and fibrosis development in mice. Fig. S7. J2 induced cross-linking activity of HSP27 as well as pHSP27. Fig. S8. Nontoxic pharmacological HSP25 inhibitor, J2 as an inhibitor of RIPF. [file 12929_2023_896_MOESM2_ESM.docx]

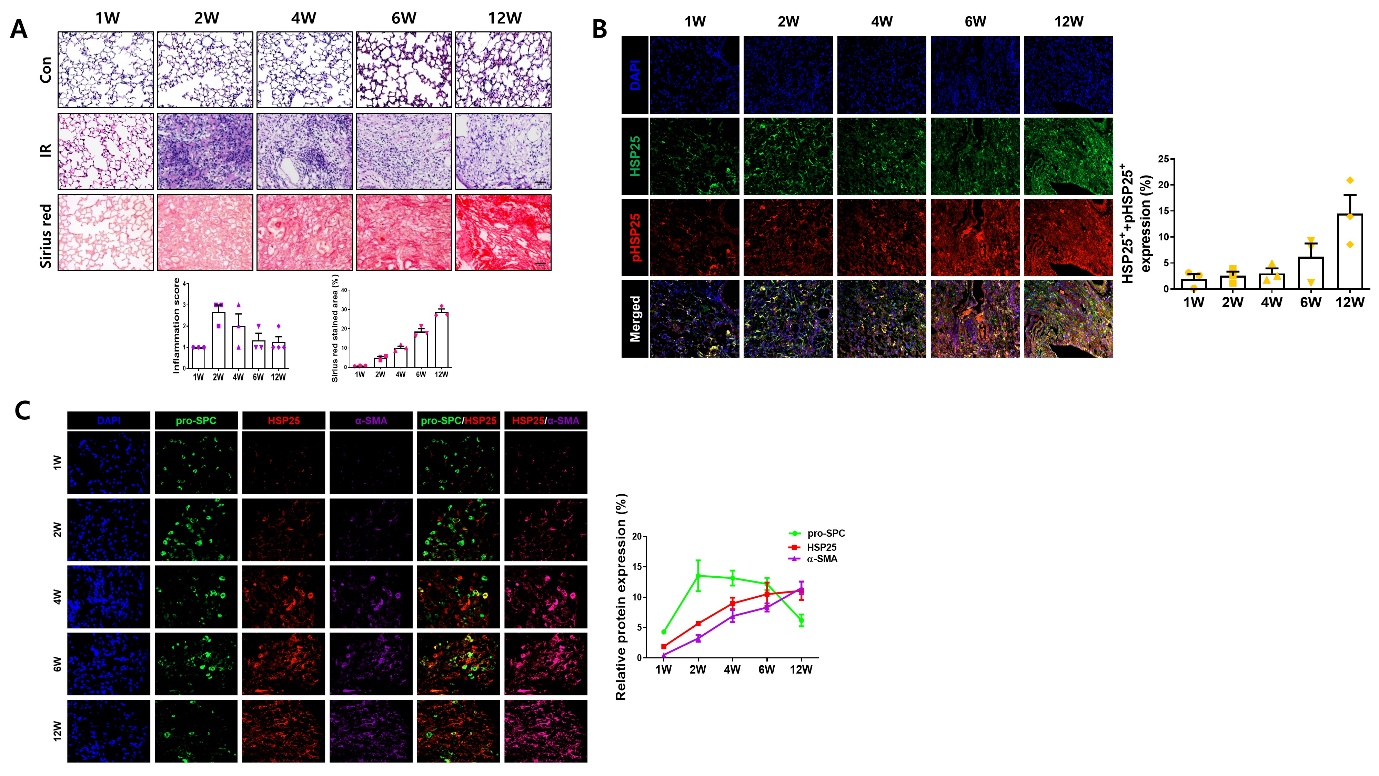

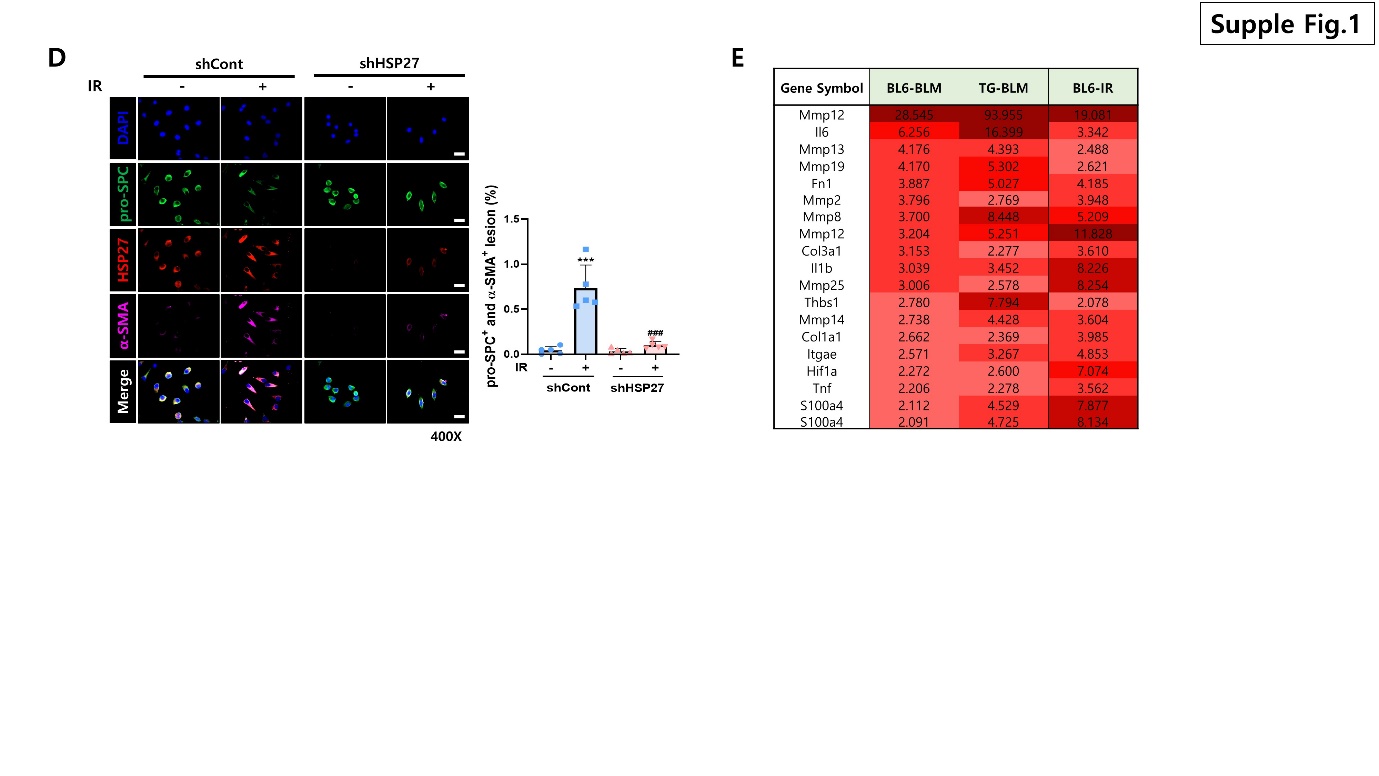


**Additional file 2: Fig. S1. Involvement of HSP27 in IR-induced EMT**

**(A)** Representative images of mouse lung sections stained with H&E and Sirius red at indicated times after focal 75 Gy irradiation (IR). **(B)** HSP25 (green) was co-stained with pHSP25 (red) using the lung tissues of indicated times after focal 75 Gy IR. The graph shows the relative levels of co-localization of HSP25 and pHSP25 as the average of 30 fields, Magnification, 200×; n=3). **(C)** Pro-SPC (green) was used for identifying type II AECs, co-stained with HSP25 (red) and α-SMA (violet). HSP25 and α-SMA expression levels were upregulated in type II AECs of the irradiated lung tissue. Magnification, 400×. Scale bar, 20 μm. **(D)** Immunofluorescence staining for pro-SPC (green), HSP27 (red), and α-SMA (violet) in L132 cell lines with a stable HSP27 knockdown at 12 h after 5 Gy IR. Magnification at 400 X, Scale bar, 20 μm. **(E)** Mice were sacrificed at 2 weeks after bleomycin intratracheal treatment or at 6 weeks after IR. The fibrotic region of the lungs was used for microarray analysis. Microarray data of fibrosis-related genes were analyzed. The list of fibrosis-related genes.


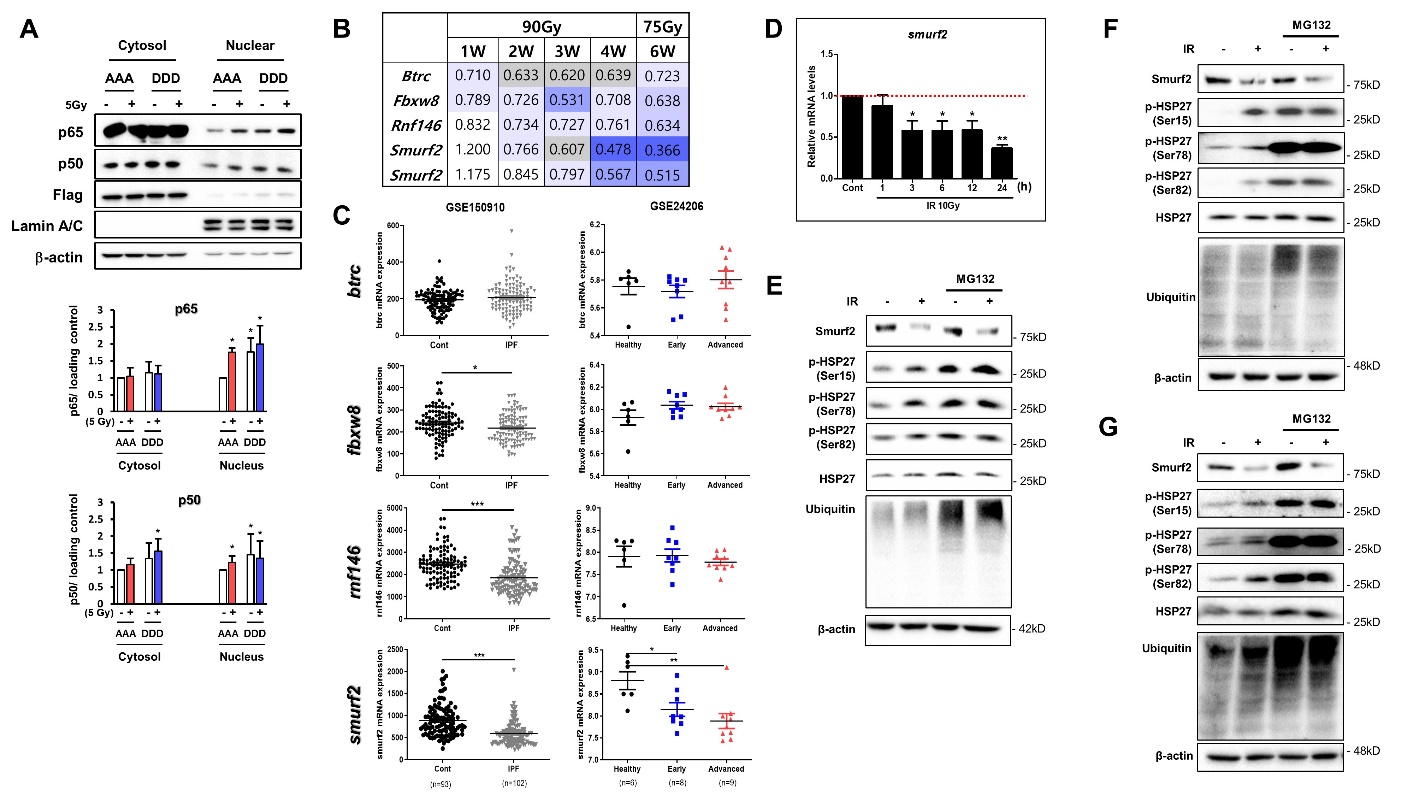

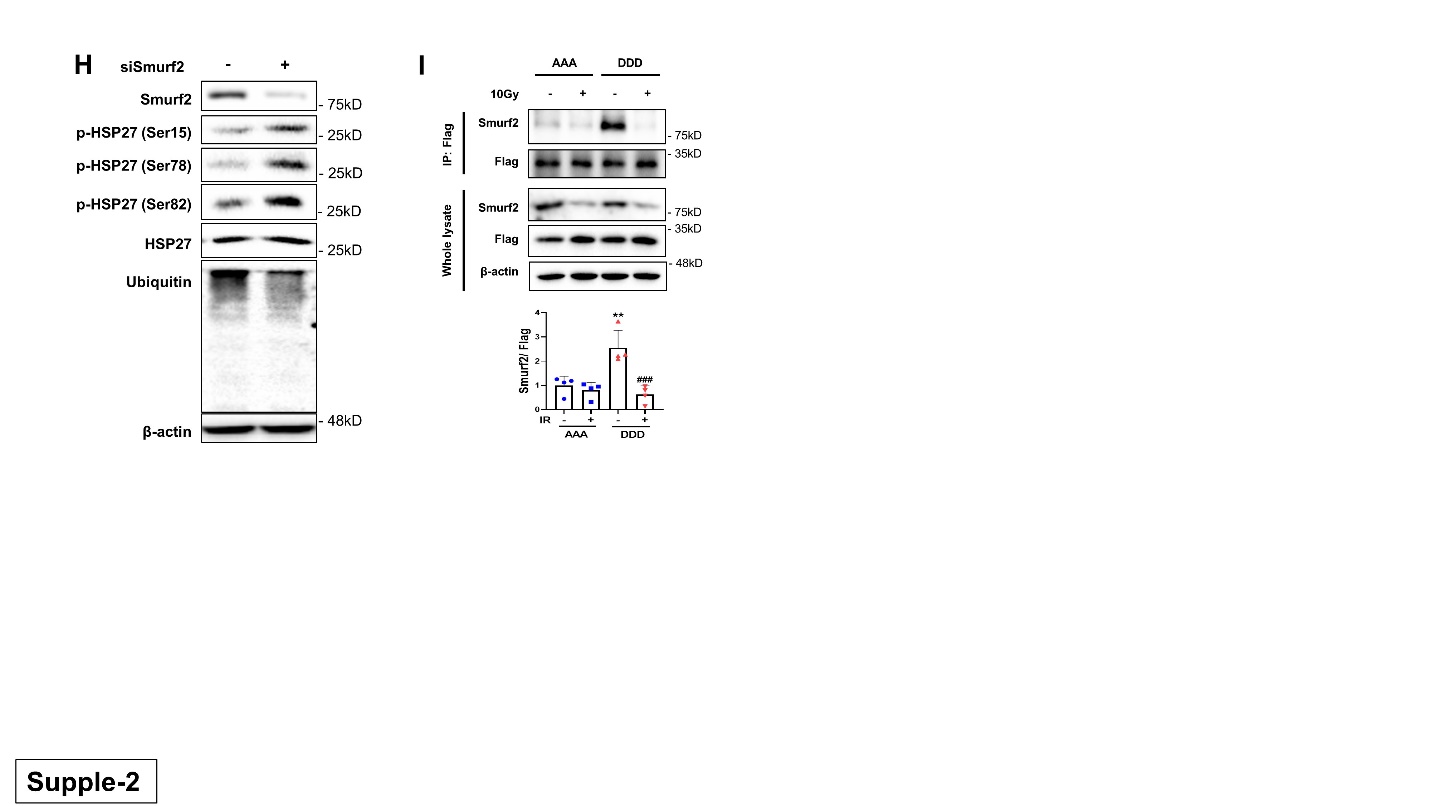


**Additional file 2: Fig. S2. Decrease expression of Smurf2 by phosphorylated HSP27**

**(A)** Western blots showed p65 and p50 translocation in irradiated cells. L132 cells were co-transfected with AAA or DDD and fractionated to yield nucleus and cytosol fractions. The purity of the fractions was verified by western blotting analysis using anti-Lamin B and anti-β-actin antibodies to detect nucleic and cytosolic proteins, respectively. **(B)** Microarray data of ubiquitin-related genes **(C)** The expression of these 4 genes in lung tissues of IPF patients using the NCBI GEO database (accession numbers GSE150910 and GSE24206). (mean±SD). *^*^P* < 0.05, *^**^P* < 0.01, and *^***^P* < 0.001 *vs*. control. **(D)** Cell lysates of L132 cells at the indicated times after 10 Gy irradiation (IR) were analyzed by quantitative RT-PCR. mRNA expression of *smurf2* was normalized to *gapdh* (n=3, mean±SD). **P* < 0.05, and ***P* < 0.005 *vs.* Control. **(E)** Western blots using cell lysates 12 h after 10 Gy IR with or without MG132 (10 μM) pre-treatment in L132 cells, displayed by representative blot and quantifications. **(F-G)** Western blots using cell lysates 12 h after 10 Gy IR with or without MG132 (10 μM) pre-treatment in human primary pulmonary fibroblast cells (F), and human primary small airway epithelial cells (G) **(H)** Western blots using cell lysates in L132 cells with siControl or siSmurf2 transfection **(I)** L132 cells were co-transfected with AAA and DDD. Cell lysates were subjected to immunoprecipitation and immunoblotting (left). The ratio of each protein to β-actin in all western blot data (right). *n* ≥ 3 biologically independent samples and results are representative of independent experiments. Data are expressed as mean±SEM. Subsequent statistical analysis was performed with one-way ANOVA with Newman-Keuls test for multiple comparisons. ***P* < 0.01 *vs.* AAA-Control; ^###^*P* < 0.001 *vs.* DDD-control.

**
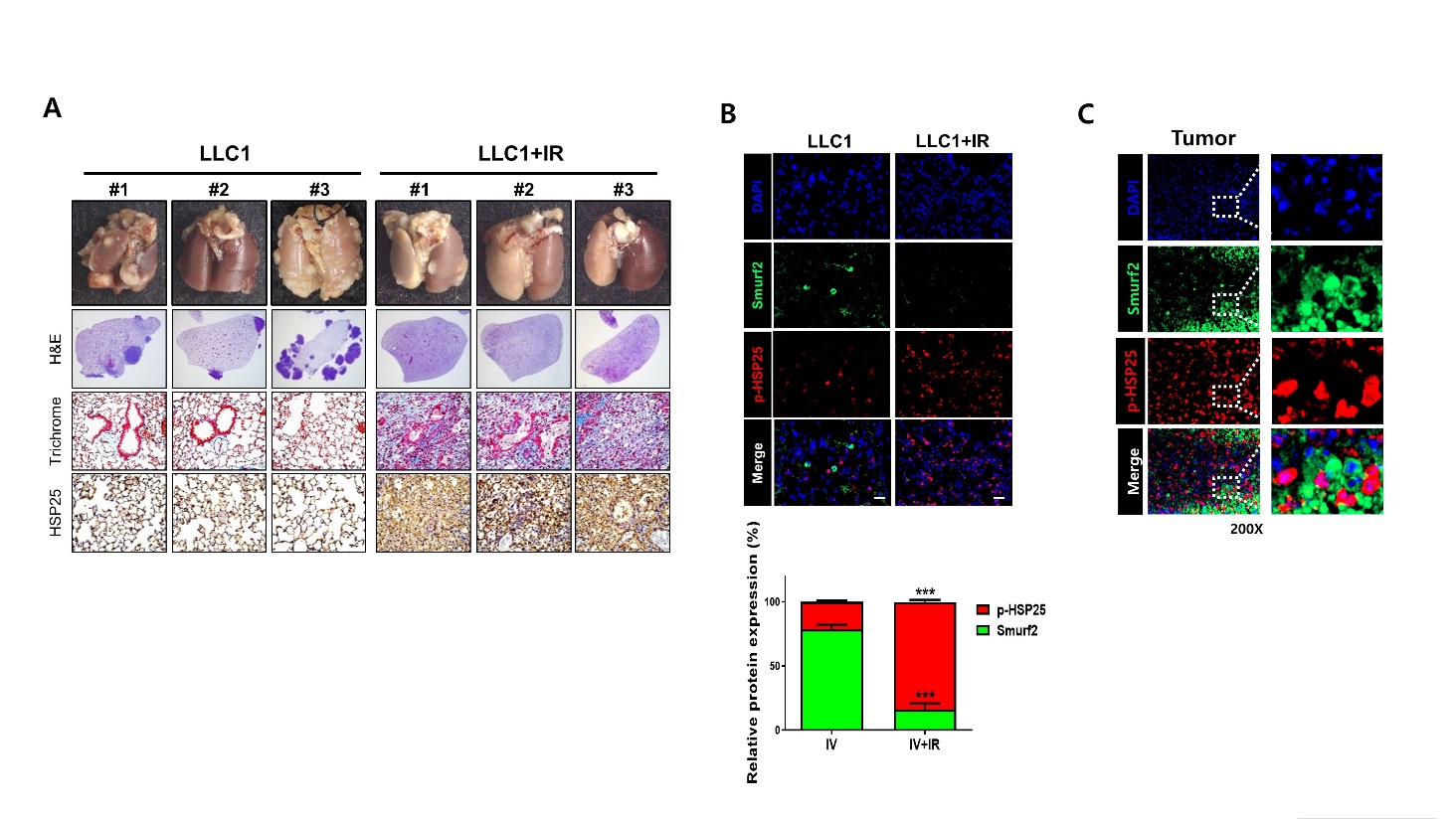
**

**Additional file 2: Fig. S3. Inverse correlation between pHSP25 and Smurf2 in irradiated orthotropic lung tumor models**

**(A)** LLC1 group: intravenous (i.v.) injection only group; LLC1+90 Gy group: mice were exposed to a single dose of 90 Gy, which was delivered to the whole left lung after 2 weeks of i.v. injection. On week 4, the mice were sacrificed and the tissues were stained with H&E, Masson’s trichrome, and HSP25. Each image was from each mouse harvested at same time point after treatment. **(B)** Smurf2 (green) was co-stained with pHSP25 (red) using the lung tissues of LLC1 and LLC1 +90Gy mice. The graph shows the relative levels of co-localization of Smurf2 and pHSP25 as the average of 30 fields, Magnification, 400×, Scale bar, 20μm; n=3). **(C)** Smurf2 (green) was co-stained with pHSP27 (red) using the tumor region of LLC1 mice.

**
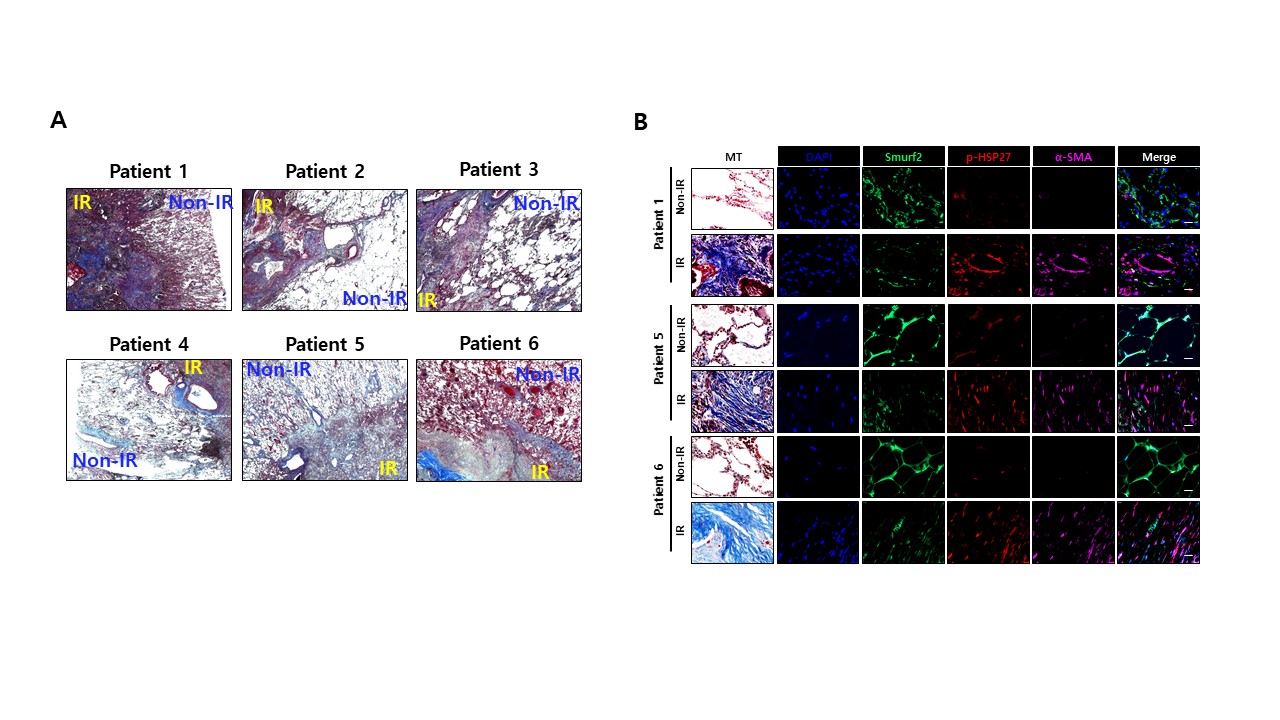
**

**Additional file 2: Fig. S4. Inverse correlation between pHSP27 and Smurf2 in RIPF patient tissues**

**(A)** Sections from human RIPF tissues were stained for Masson’s trichrome. Magnification, 12.5×. **(B)** The tissues were stained with Smurf2 (green), pHSP27 (Red), and α-SMA (violet). Magnification, 400×. Scale bar, 20 μm.

**
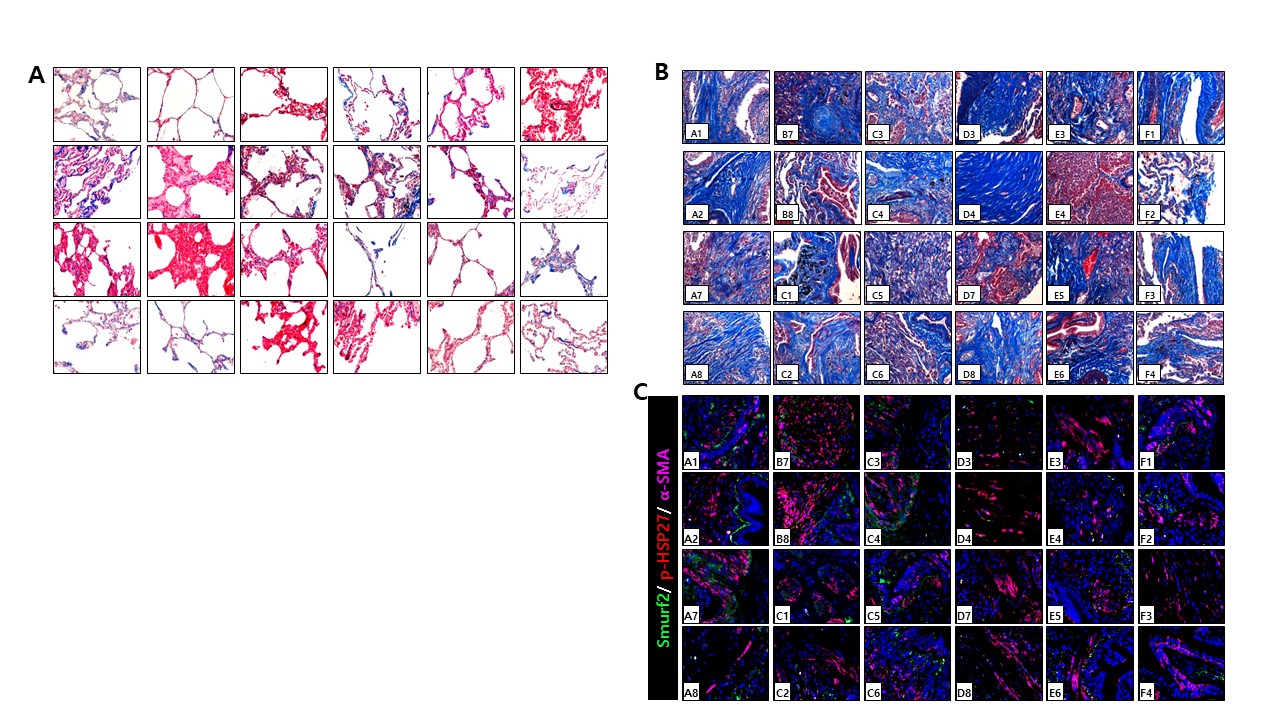
**

**Additional file 2: Fig. S5. Inverse correlation between pHSP27 and Smurf2 in IPF patient tissues**

**(A)** Sections from human normal lung tissues were stained for Masson’s trichrome. Magnification, 200×. **(B)** Sections from human IPF patient lung tissues were stained for Masson’s trichrome. Magnification, 200×. **(C)** The tissues were stained with Smurf2 (green), pHSP27 (Red), and α-SMA (violet). Magnification, 400×.

**
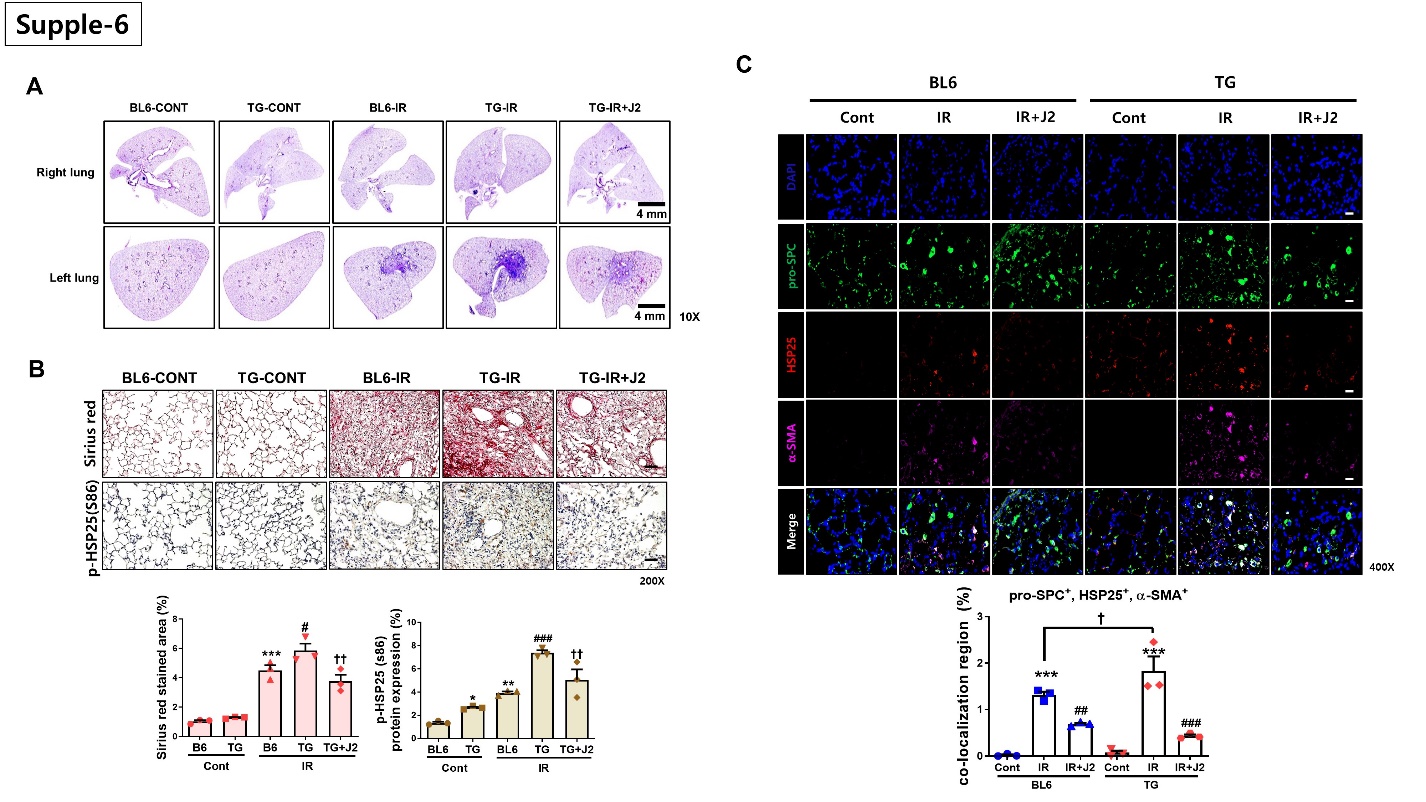
**

**Additional file 2: Fig. S6. HSP25 cross-linker J2 inhibited IR-induced EMT and fibrosis development in mice.**

C57BL6N (BL6) and HSP25 transgenic (TG) mice were sacrificed at 6 weeks after 75 Gy irradiation (IR). The mice were intra-peritoneally administered with J2 (15 mg/kg) on alternate days after IR for 4 weeks. On week 6, the mice were sacrificed. **(A)** Haematoxylin & eosin staining was performed. Magnification, 10×, Scale bar, 4mm. **(B)** The lungs were stained with Sirius red staining (top) and pHSP25 (ser86) (bottom). Graphs represent the quantification of the positive region. Magnification, 200X, Scale bar, 100um. ; n=3, mean±SEM; **P* < 0.05, ***P* < 0.01 and ****P* < 0.001 vs. BL6^_^Control; *^#^P* < 0.05 and *^###^P* < 0.001 vs. BL6^_^IR; *^††^P* < 0.01 vs. TG- IR. **(C)** Pro-SPC (green) was co-stained with HSP25 (red) and α-SMA (violet) using lung tissues of BL6 and HSP25 TG. HSP25 and α-SMA expression levels were upregulated in type II AECs of the irradiated lung tissue. Magnification, 400×. Scale bar, 20 μm. Quantification of pro-SPC, HSP25 and α-SMA positive cell was presented as mean ± SEM (****P* < 0.001 vs. each Control group; ^##^*P* < 0.01 and ^###^*P* < 0.001 vs. each IR group; ^†^*P* < 0.05 vs. B6-IR).


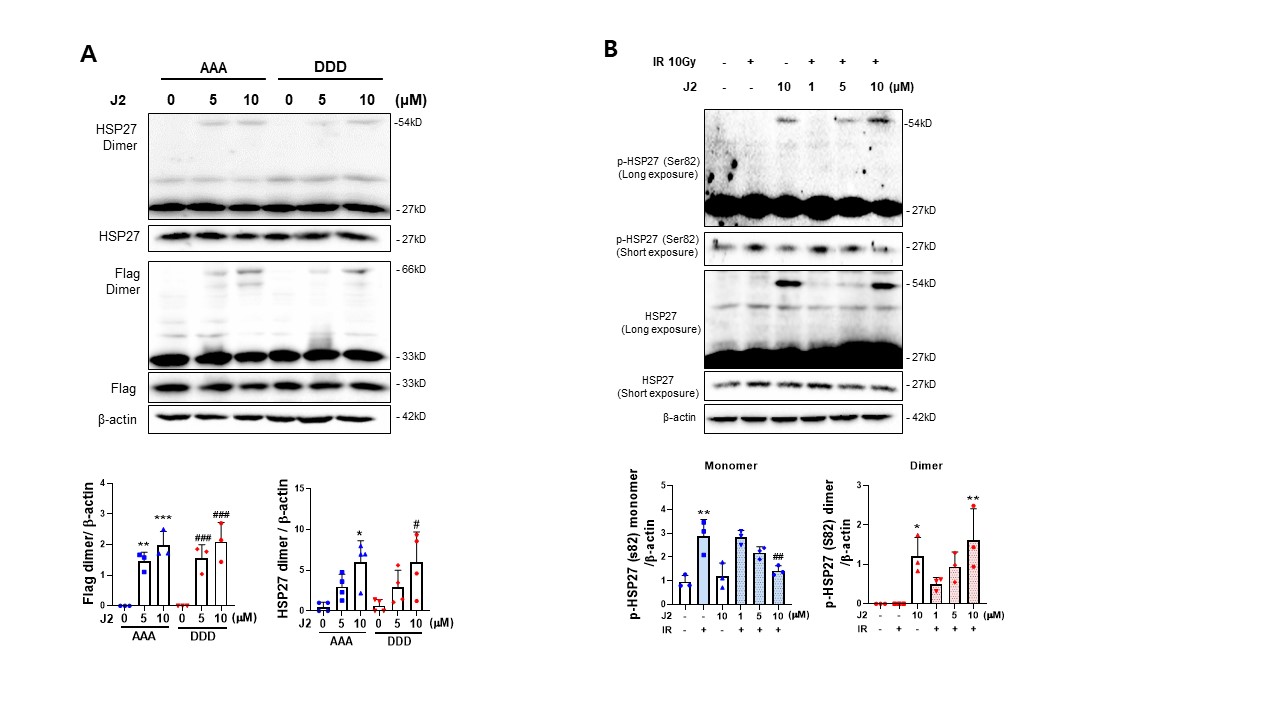


**Additional file 2: Fig. S7. J2 induced cross-linking activity of HSP27 as well as pHSP27.**

**(A)** L132 cells were transfected with AAA or DDD and treated with J2 (0, 5, 10 μM). n ≥ 3, mean±SEM; **P* < 0.05, ***P* < 0.01 and ****P* < 0.001 vs. AAA^_^Control; *#P*< 0.05 and *###P* < 0.001 vs. DDD^_^Control_**(B)** L132 cells were treated with J2 (0, 1, 5, and 10 μM) or 10 Gy IR for 24 h. Cell lysates were detected and displayed by representative blot and quantifications. **P* < 0.05 and ***P* < 0.01 vs. Control; *##P* < 0.01 vs. IR only. Ratio of each protein to β^_^actin in all western blot data. *n*≥ 3 biologically independent samples and results are representative of independent experiments. Data are expressed as mean±SD. Subsequent statistical analysis was performed with one^_^way ANOVA with Newman^_^Keuls test for multiple comparisons.

**
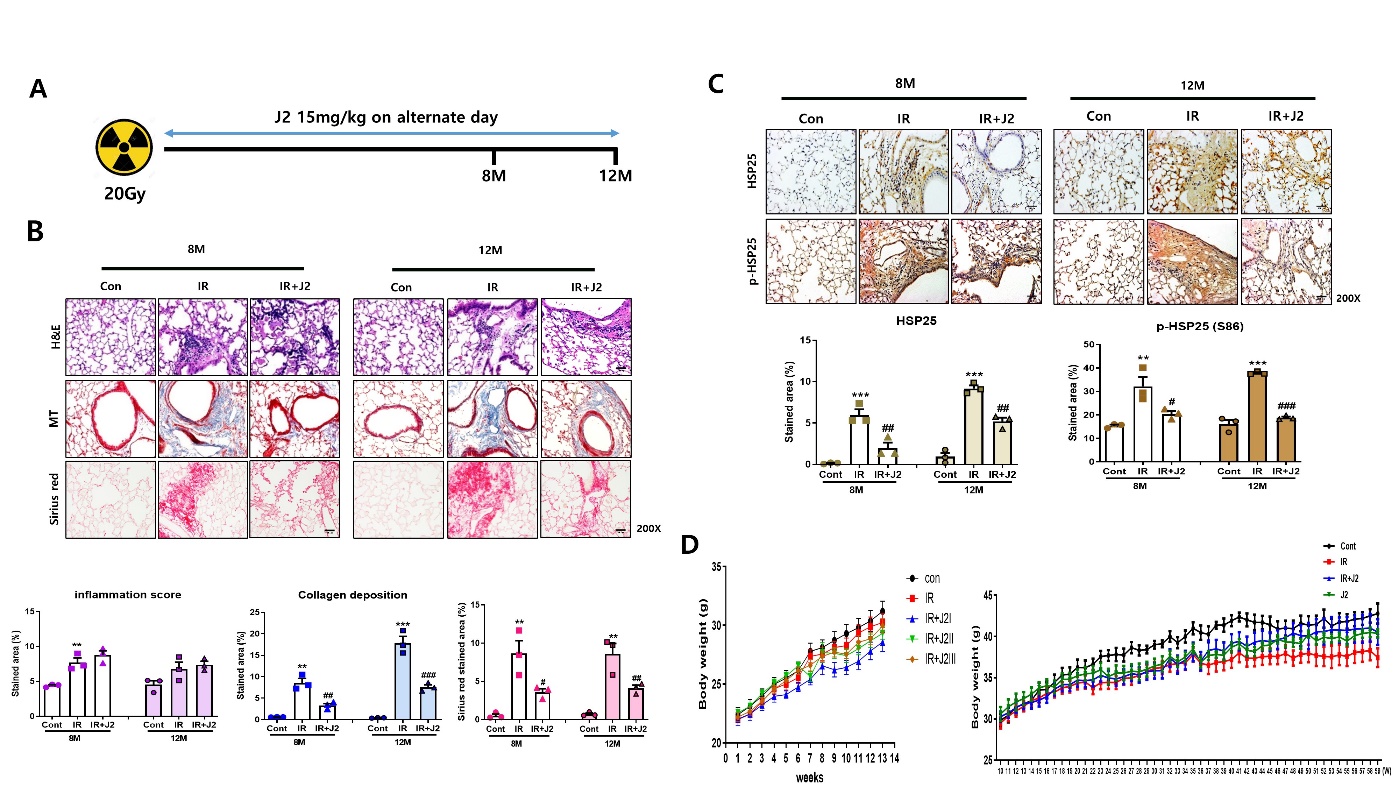
**

**Additional file 2: Fig. S8.** **Nontoxic pharmacological HSP25 inhibitor, J2 as an inhibitor of RIPF.**

Mice were sacrificed at the indicated times after 20 Gy irradiation (IR). The mice were intra-peritoneally administered with J2 (15 mg/kg) on alternate days after IR. On 8 or 12 months, the mice were sacrificed. **(A)** Experimental scheme of focal exposure to low-dose radiation (20 Gy) to mice by periods. **(B)** Lungs were stained with H&E (top), Masson’s trichrome (middle), and Sirius red (bottom). The graph shows the quantification of the stained region. *n* ≥ 3, mean±SEM; ***P* < 0.01, ****P* < 0.001 *vs*. Control; ^#^*P* < 0.05, ^##^*P* < 0.01, and ^###^*P* < 0.001 *vs*. IR only. **(C)** Immunohistochemistry of HSP25 (top) and pHSP25 (Ser 86) (bottom) in mouse lung tissues was performed. The graph shows the quantification of positive cells. *n* ≥ 3, mean±SEM; ***P* < 0.01, ****P* < 0.001 *vs*. Control; ^#^*P* < 0.05, ^##^*P* < 0.01, and ^###^*P* < 0.001 *vs*. IR only. **(D)** Body weight changes in 75 Gy, 13 weeks mice model (left) and in 20 Gy, 12 months mice model (right).
